# LONP1 facilitates pulmonary artery smooth muscle cell glycolytic reprogramming by degrading MPC1 in pulmonary hypertension

**DOI:** 10.1042/CS20255922

**Published:** 2025-05-20

**Authors:** Mingkang Li, Wenkang Zhang, Minhao Zhang, Linqing Li, Yuyu Yao, Yuhan Qin, Dong Wang, Gaoliang Yan, Yong Qiao, Chengchun Tang

**Affiliations:** 1 Department of Cardiology, Zhongda Hospital, Southeast University, 87 Dingjiaqiao, Nanjing, Jiangsu 210009, China; 2 School of Medicine, Southeast University, Nanjing, Jiangsu, China; 3 Department of Cardiology, Nanjing First Hospital, Nanjing Medical University, Nanjing, Jiangsu, China

**Keywords:** glycolytic reprogramming, Lon protease 1, mitochondrial pyruvate carrier 1, pulmonary hypertension, pulmonary vascular remodeling

## Abstract

Pulmonary hypertension (PH) is a chronic and life-threatening disease characterized by pulmonary vascular remodeling (PVR), which involves the abnormal proliferation of pulmonary artery smooth muscle cells (PASMCs). These cells exhibit metabolic characteristics akin to cancer cells, particularly in their shift toward glycolysis. The Lon protease 1 (LONP1) has been shown to promote glycolytic reprogramming of tumor cells, conferring a malignant proliferative phenotype. However, the precise role of LONP1 in PH remains unclear. In the present study, Su5416/hypoxia-induced and monocrotaline (MCT)-induced PH rodent models and platelet-derived growth factor BB (PDGF-BB)-induced PASMCs were used to investigate the role and mechanism of LONP1 in PH. The results revealed an up-regulation of LONP1 expression in lung tissues from two PH rodent models, as well as in PDGF-BB-induced PASMCs. *In vivo* knockdown of LONP1 significantly alleviated PASMC mitochondrial dysfunction, reduced glycolytic enzyme expression, and decreased lactate accumulation, thereby mitigating PVR. Additionally, *in vitro* experiments demonstrated that knockdown or inhibition of LONP1 attenuated glycolytic reprogramming, proliferation, and migration of PASMCs, whereas overexpression of LONP1 had converse effects. Mechanistic studies confirmed that mitochondrial pyruvate carrier 1 (MPC1) was a direct substrate for LONP1-mediated degradation. Functional experiments with MPC1 knockdown and overexpression further elucidated its role in the proliferation and migration of PASMCs. Rescue experiments indicated that MPC1 knockdown abrogated the suppressive effects of LONP1 knockdown on glycolytic reprogramming, proliferation, and migration in PASMCs. Therapeutically, knockdown or pharmacological inhibition of LONP1 significantly reversed MCT-induced PH in rats. Thus, targeting LONP1 may represent a promising therapeutic strategy for PH.

## Introduction

Pulmonary hypertension (PH) is a chronic and fatal cardiopulmonary disease, characterized by pulmonary vascular remodeling (PVR) and sustained elevation of pulmonary artery pressure, prominently resulting from significant thickening of the pulmonary arteriolar walls and luminal narrowing [1]. The primary forces driving PVR are the abnormal proliferation, migration, and increased resistance to apoptosis of pulmonary artery smooth muscle cells (PASMCs) [2]. Current therapeutic strategies for PH primarily involve medications such as endothelin receptor antagonists, prostacyclins, phosphodiesterase-5 inhibitors, and soluble guanylate cyclase activators. Despite alleviating clinical symptoms to some extent, these pharmacological agents have not significantly reduced PH-related mortality [3,4]. Consequently, there is an urgent need to delve deeper into the molecular mechanisms of PH to provide novel insights for the development of more effective clinical treatments.

Accumulating evidence suggests that metabolic reprogramming, particularly alterations in glucose metabolism, plays a pivotal role in the pathogenesis of PH [5]. During this abnormal metabolic process, mitochondrial dysfunction shifts glucose metabolism from mitochondrial oxidative phosphorylation (OXPHOS) to cytosolic glycolysis. This metabolic shift accelerates glucose uptake and utilization to meet the energy demands of hyperproliferative, tumor-like cells, akin to the Warburg effect observed in cancer [6]. Studies demonstrated a significant increase in fluorodeoxyglucose uptake in the lungs of patients with idiopathic pulmonary artery hypertension, confirming enhanced glycolytic activity during PH [7,8]. Additionally, enhanced glycolysis induced phosphorylation of extracellular signal-regulated kinases and activation of calpain, thereby promoting the proliferation of PASMCs [9]. Importantly, targeting the key enzymes in glucose metabolism has been shown to reverse hypoxia-induced glycolytic reprogramming in PASMCs, thereby ameliorating PVR [10,11]. These findings emphasize that inhibiting glycolytic reprogramming is a potentially effective therapeutic strategy for PH; however, the underlying mechanisms triggering glycolytic reprogramming in PASMCs require further elucidation.

Lon protease 1 (LONP1) is a highly conserved adenosine triphosphate (ATP)-dependent mitochondrial protease primarily located in the mitochondrial matrix. It comprises an N-terminal substrate recognition domain, an AAA^+^ module for ATP binding and hydrolysis, and a C-terminal domain containing the proteolytic active site [12]. LONP1 not only specifically binds to and degrades abnormal proteins but also participates in the regulation of critical processes such as mitochondrial DNA replication and biosynthesis, playing a pivotal role in maintaining mitochondrial protein homeostasis, cellular stress responses, and metabolism [13]. A homozygous deletion of LONP1 results in embryonic lethal, while a reduction in LONP1 expression or activity leads to abnormal accumulation of mitochondrial proteins, inhibiting cell proliferation and inducing apoptosis, potentially triggering a range of diseases including renal fibrosis, bone metabolic disorders, neuronal dysfunction, and oocyte developmental abnormalities [14–18]. Conversely, studies have found increased expression of LONP1 in various cancer tissues, correlating with poor patient survival rates [19]. LONP1 promoted tumor cell proliferation and invasion by remodeling mitochondrial electron transport chain (ETC) complexes to mediate glycolytic reprogramming. Knockdown of LONP1 reduced tumor growth and metastasis *in vivo*, whereas overexpression enhanced them [15]. Furthermore, overexpression of the LONP1 mitigated liver injury by restoring the expression and enhancing the activity of critical gluconeogenic enzymes [20]. Additionally, this overexpression reversed insulin resistance and enhanced glucose tolerance in murine models of diabetes [21]. Bioinformatics analysis elucidated a significant positive correlation between increased LONP1 expression and the up-regulation of genes implicated in glucose and lipid metabolism [22]. However, the role and underlying mechanisms of LONP1 in PH development remain poorly understood.

In the present study, a significant up-regulation of LONP1 expression was observed in PH. *In vivo*, LONP1 knockdown substantially mitigated monocrotaline (MCT)-induced PVR and reduced pulmonary artery pressure. Subsequent experiments further clarified that LONP1 facilitated glycolytic reprogramming, proliferation, and migration of PASMCs by degrading mitochondrial pyruvate carrier 1 (MPC1). Notably, knockdown or inhibition of LONP1 reversed the established PH phenotype in rats. These findings suggest that LONP1 in PASMCs may represent a novel therapeutic target for PH.

## Methods

### Animal models

C57BL/6 J mice (6–8 weeks old) and Sprague-Dawley (SD) rats (5–6 weeks old) were acquired from the Animal Center of Southeast University and housed in facilities free of pathogens. Due to the estrogen paradox in PH, only male animals were included in this study to eliminate the effect of estrogen on PH and reduce experimental variability [23]. All experiments were conducted following the Guide for the Care and Use of Laboratory Animals published by the National Institutes of Health and were approved by the Animal Experimentation Ethics Committee of Southeast University (Approval number: 20240314002). All animals were maintained under controlled photocycle (12 hours light/12 hours dark) and temperature (22–25°C) with *ad libitum* access to food. Animal experiments were conducted at the Animal Center of Southeast University. After experiments, the animals were anesthetized by inhaling isoflurane and then killed by cervical dislocation.

Two PH models were utilized to investigate LONP1 expression: (1) Su5416/hypoxia (SuHx)-induced PH in mice: after acclimatization, the animals were randomly assigned to a control group (*n* = 7) and a SuHx group (*n* = 7). Control mice received subcutaneous injections of the vehicle and were maintained in normoxic conditions. SuHx mice were exposed to chronic hypoxia (10% O_2_) for four weeks with weekly subcutaneous injections of Su5416 (20 mg/kg; S2845, Selleck Chemicals, Houston, U.S.A.). (2) MCT-induced PH in rats: after acclimatization, the animals were randomly assigned to a control group (*n* = 5) and an MCT group (*n* = 5). Control rats received injections of an equivalent volume of saline. MCT rats received a single intraperitoneal injection of MCT (60 mg/kg; HY-N0750, MedChemExpress, Shanghai, China), which induced PH after three weeks. Hemodynamics, ventricular function, and pathology were assessed in both mouse and rat models, and lung tissue samples were collected for subsequent experiments.

LONP1 knockdown or inhibition experiments were conducted to evaluate its preventive and therapeutic effects on PH. The serotype 9 adeno-associated virus (AAV) carrying a smooth muscle 22 alpha (SM22α) promoter and shRNA sequence specific for rat LONP1 (pAAV-SM22ap-miR30shLonp1-WPRE, AAV-shLONP1) or negative control (pAAV-SM22ap-miR30shNC-WPRE, AAV-shNC) were constructed by the OBiO technology company (Shanghai, China). In the study assessing the preventive effect of LONP1 knockdown, rats were randomly assigned to the AAV-shNC group (*n* = 6), AAV-shLONP1 group (*n* = 6), AAV-shNC + MCT group (*n* = 8), and AAV-shLONP1 + MCT group (*n* = 8). Two weeks before MCT (60 mg/kg) injection, AAV-shNC and AAV-shLONP1 (100 μl per rat, 3.5 × 10^12^ vg/ml) were administered via intratracheal injection. In the study assessing the therapeutic effect of LONP1 knockdown or inhibition, rats were randomly assigned to the control group (*n* = 5), MCT group (*n* = 15), MCT + AAV-shLONP1 group (*n* = 10), and MCT + bortezomib (BTZ) group (*n* = 10). Three weeks after the injection of MCT (50 mg/kg), either a single intratracheal injection of AAV-shLONP1 (100 μl per rat, 3.5 × 10^12^ vg/ml) or intraperitoneal injection of BTZ (100 μg/kg; HY-10227, MedChemExpress, China) administered twice a week was used. Three weeks later, evaluations of hemodynamics, ventricular function, and pathology were conducted, and lung tissue samples were collected for subsequent experiments.

### Echocardiographic assessment

Experimental animals were anesthetized in an induction chamber with continuous isoflurane inhalation, while the ambient temperature was maintained at approximately 25°C. Following the removal of chest hair, transthoracic echocardiographic evaluations were conducted by a skilled operator utilizing a Visual Sonics Vevo 2100 ultrasound system equipped with a 30 MHz probe to assess cardiac functionality. Pulmonary artery acceleration time (PAT), pulmonary artery ejection time (PET), PAT/PET ratio, and tricuspid annular plane systolic excursion (TAPSE) were objectively measured by a blinded echocardiography specialist.

### Assessment of hemodynamic parameters and Fulton index

Experimental animals were anesthetized through isoflurane inhalation and then underwent a closed-chest procedure for the insertion of a catheter, which was connected to a pressure sensor and positioned in the right ventricle (RV) to measure RV systolic pressure (RVSP). The MPA cardiac function analysis system (Alcott Biotech, Shanghai, China) was utilized for the collection and analysis of hemodynamic waveforms. Upon killing, the hearts of the animals were excised and subsequently rinsed with physiological saline. The RV, left ventricle (LV), and septum (S) were carefully dissected and weighed to calculate the Fulton index. The Fulton index was derived by dividing the weight of RV by the sum of the weights of the LV and S.

### Hematoxylin and eosin staining, Masson’s trichrome stain, immunohistochemistry, and immunofluorescence staining

Animal tissues were fixed at room temperature using 4% paraformaldehyde (PFA), embedded in paraffin, and sectioned into 5-μm-thick slices, which were subsequently dewaxed. Hematoxylin and eosin (H&E) staining was employed for detailed morphological analysis of pulmonary arteries and hearts. PVR was quantified by measuring pulmonary artery wall thickness, calculated as the ratio of (total vascular area minus luminal area) to total vascular area. Masson’s trichrome stain kit (G1006, Servicebio, Wuhan, China) was used to stain heart sections for the evaluation of cardiac fibrosis. For immunohistochemistry (IHC), antigen retrieval was conducted on the sections using citrate buffer, followed by treatment with a 3% hydrogen peroxide solution to inactivate endogenous peroxidase activity. After thorough washing with phosphate-buffered saline (PBS), the sections were sequentially incubated with the primary antibody [rabbit anti-LONP1 (1:300, 15440–1-AP, Proteintech, Wuhan, China)], the corresponding secondary antibody, and diaminobenzidine for visualization. For immunofluorescence (IF) staining, the sections were permeabilized using a permeabilization buffer and blocked with 5% bovine serum albumin (BSA) at room temperature for 1 hour. They were then incubated overnight at 4°C with a cocktail of primary antibodies, including rabbit anti-LONP1 (1:500, 15440–1-AP, Proteintech), rabbit anti-MPC1 (1:100, A20195, ABclonal, Wuhan, China), and rabbit anti-α-smooth muscle actin (α-SMA) (1:2000, 14395–1-AP, Proteintech). Following incubation, the slides were washed and incubated with the secondary antibody at room temperature for 2 hours. Subsequently, the nuclei were stained with 4’,6-diamidino-2-phenylindole (DAPI) (BL105A, Biosharp, Hefei, China). Image acquisition was performed using a digital slide scanner (vs.200, OLYMPUS, Tokyo, Japan).

### Transmission electron microscopy

Transmission electron microscopy (TEM) was utilized to confirm the mitochondrial integrity within the smooth muscle layer of rat pulmonary arteries. The lung tissues underwent a meticulous process: initial fixation with 3% glutaraldehyde, followed by refixation in 1% osmium tetroxide, and progressive dehydration using acetone. Afterward, the tissues were embedded in an Epon-812 embedding medium. Through semi-thin sectioning, all samples were examined under a light microscope, allowing for the selection of the smooth muscle layer of rat pulmonary arteries. Using an ultra-microtome, ultra-thin sections (ranging from 60 to 90 nm in thickness) were obtained and transferred onto copper grids. These grids were then stained with uranyl acetate and lead citrate to enhance visualization. Finally, images of the stained copper grids were captured using the TEM (JEM-1400FLASH, JEOL, Tokyo, Japan).

### Isolation and culture of primary rat PASMCs

Primary rat PASMCs were isolated from male SD rats (120–160 g) as previously described [24]. The isolation procedure began with the euthanasia of the rats, followed by skin disinfection using 75% ethanol. The chest cavity was then opened to expose and excise the heart and lungs, which were subsequently rinsed with pre-chilled PBS. In a sterile setting, the pulmonary arteries were dissected free of extraneous tissues. The vessels were longitudinally opened, and the endothelial cells were gently removed by scraping. The cleaned pulmonary arteries were then minced into 1 mm³ tissue fragments. These fragments were cultured using the physical adherence method until cell confluence, at which point they were passaged. The identity of the PASMCs was verified through immunostaining with α-SMA (1:2000, 14395–1-AP, Proteintech). The cells were maintained in high-glucose Dulbecco’s modified eagle medium (DMEM) (Gibco, New York, U.S.A.), supplemented with 10% fetal bovine serum (FBS) and 1% penicillin-streptomycin. Culturing was performed in an incubator maintained at 37°C, with 5% CO_2_ and saturated humidity. All experiments utilized PASMCs from passages 2 to 4.

### Cellular transfection

Small interfering RNA (siRNA) sequences designed to knock down LONP1 expression, lentiviral vectors engineered for LONP1 overexpression (Lenti-LONP1), and their respective negative controls were synthesized by GenePharma Biotechnology (Shanghai, China). Analogously, siRNA sequences designed to knock down MPC1 expression, plasmids constructed for MPC1 overexpression (OE-MPC1), and their corresponding negative controls were synthesized by Gene Universal (Anhui, China). Cellular transfection was performed following the manufacturer’s instructions. Subsequent experiments were initiated after verifying the knockdown and overexpression efficiencies of the target genes.

### Western blot analysis

Total protein was extracted utilizing a total protein extraction kit (KGP2100, Keygen biotech, Nanjing, China), followed by determination of the protein concentration using a bicinchoninic acid (BCA) protein assay kit (BL521A, Biosharp). Identical quantities of protein were loaded onto sodium dodecyl sulfate-polyacrylamide gel electrophoresis (SDS-PAGE) gels for separation and then transferred onto polyvinylidene fluoride membranes. The membranes were blocked with 5% non-fat milk at room temperature for 1.5 hours. Then, the membranes were incubated overnight at 4°C with specific antibodies including rabbit anti-LONP1 (1:4000, 15440–1-AP, Proteintech), rabbit anti-MPC1 (1:1000, A20195, ABclonal), rabbit anti-MPC2 (1:1000, 20049–1-AP, Proteintech), rabbit anti-Hexokinase 2 (HK2) (1:30000, 22029–1-AP, Proteintech), rabbit anti-glucose transporter-1 (GLUT1) (1:4000, 21829–1-AP, Proteintech), rabbit anti-lactate dehydrogenase A (LDHA) (1:5000, 19987–1-AP, Proteintech), rabbit anti-proliferating cell nuclear antigen (PCNA) (1:10000, 10205–2-AP, Proteintech), and mouse anti-β-actin (1:10000, 20536–1-AP, Proteintech). Subsequently, the membranes were washed and incubated with the respective secondary antibodies (1:10000, SA00001-1 or SA00001-2, Proteintech) for 1 hour. Ultimately, the protein bands were visualized through autoradiography, and the housekeeping protein (β-actin) normalization method was employed for relative quantitative analysis of protein expression. Briefly, the signals of the target protein and β-actin in all lanes were quantified using ImageJ software. Then, the optical density ratio of the target protein to β-actin was computed to derive the relative optical density value. The relative optical density value of the target protein in the control group was set to 1, and the fold change of the target protein bands in the experimental groups relative to the control group was calculated.

### RNA extraction and quantitative real-time polymerase chain reaction

Total RNA was isolated from lung tissues and PASMCs by employing the TRIzol extraction reagent (R401-01, Vazyme, Nanjing, China). Following this, the RNA was reverse-transcribed into cDNA utilizing a commercial kit (R323, Vazyme). Quantitative real-time polymerase chain reaction (qPCR) was performed on the CFX96 BIOER™ Real-Time PCR System (Hangzhou, China) using Taq Pro Universal SYBR qPCR Master Mix (Q712, Vazyme), adhering strictly to the manufacturer’s protocol. The relative expression levels of the target genes were normalized to β-actin expression and were quantitated by employing the 2^(-ΔΔCT) method. The primer sequences utilized for reverse transcription qPCR are presented in Table 1.

**Table 1:** Primer sequences used for qPCR.

Gene	Forward primer sequences (5’-3’)	Reverse primer sequences (5’-3’)
For mice		
LONP1	TGGCAGCAAGGAGGACAAGG	TCAGGAAGGCACGAGCATAGG
β-actin	ACTGCCGCATCCTCTTCCTC	AACCGCTCGTTGCCAATAGTG
For rats		
LONP1	TGGCAGCAAGGAGGACAAGG	AGGAAGGCACGGGCAAAGG
MPC1	CGCCCTCTGTTGCTATTCTCTGAC	CCTGAATGAGCTGAGCGACTTCG
β-actin	CTATCGGCAATGAGCGGTTCC	GCACTGTGTTGGCATAGAGGTC

### Cell counting kit-8 and EdU incorporation assays

Cell viability was assessed using the cell counting kit-8 (CCK-8) assay (BS350B, Biosharp). Briefly, PASMCs (8.0 × 10^3^/well) undergoing various treatments were plated onto 96-well plates. CCK-8 reagent was then added, and the cells were incubated at 37°C for 2 hours. Following this incubation, the absorbance at 450 nm was measured using a microplate reader (CMax Plus, Molecular Devices, San Jose, U.S.A.). To evaluate cell proliferation, EdU incorporation was conducted using the BeyoClick™ EdU Cell Proliferation Kit with Alexa Fluor 594 (C0078S, Beyotime, Shanghai, China), with strict adherence to the manufacturer’s protocol.

### Transwell assay

Cell migration was assessed using 24-well Transwell plates with an 8 μm pore size (Corning, New York, U.S.A.). PASMCs (2.0 × 10^4^/well), which had undergone various treatments, were plated in the upper chamber containing 0.1% FBS medium, whereas the lower chamber was supplemented with 10% FBS. Following a 24 hour incubation period, the cells were fixed, washed, stained, and subsequently air-dried. Microscopic examination and photographic documentation were then conducted.

### Wound healing assay

PASMCs (5.0 × 10^4^/well) were evenly distributed into a six-well plate. After various interventions, three horizontal reference lines were inscribed on the bottom of the plate. A 200 μl pipette tip was utilized to create two perpendicular scratches across the plate. Non-adherent cells were rinsed away, and the scratched area was monitored by observation and photography every 12 hours to assess wound healing progression. Migration was subsequently quantified as the percentage reduction in the scratch area using ImageJ software.

### Cell IF staining

PASMCs subjected to various interventions were fixed using 4% PFA, subsequently washed with PBS, permeabilized with 0.1% Triton X-100, blocked with 5% BSA, and then washed again with PBS. The samples were incubated overnight at 4°C with specific antibodies including mouse anti-LONP1 (1:500, 66043–1-Ig, Proteintech) and rabbit anti-MPC1 (1:100, A20195, ABclonal). For MitoTracker staining, live cells were stained with a 100 nM working solution of MitoTracker Green (C1048, Beyotime) or MitoTracker Red (C1049B, Beyotime) in confocal dishes for 20 minutes at 37°C. As required, cells were fixed, permeabilized, blocked, and incubated overnight at 4°C with the corresponding primary antibody, followed by incubation with a fluorescent secondary antibody for 1 hour at room temperature. The cell nuclei were stained with Hoechst 33,342. Representative confocal images were acquired, and mitochondrial morphology analysis was conducted using ImageJ software.

### Measurement of lactate and ATP concentrations

Lactate concentrations in cellular supernatants and animal sera were quantified utilizing a lactate assay kit (A019-2-1, Jiancheng, Nanjing, China), adhering strictly to the manufacturer’s protocol. In brief, the cellular supernatants or sera were diluted with physiological saline and subsequently mixed with enzyme working solution and chromogenic agent. Following thorough agitation, the samples were incubated in a water bath maintained at 37°C for 10 minutes. Thereafter, a stop solution was introduced, and the absorbance of the resultant mixture was assessed at a wavelength of 530 nm using a microplate reader. Furthermore, ATP concentrations in pulmonary artery homogenates and cellular lysates were determined using an enhanced ATP detection kit (S0027, Beyotime), following the manufacturer’s guidelines.

### Energy metabolism analysis

Oxygen consumption rate (OCR) and extracellular acidification rate (ECAR) were measured using the XF-96 extracellular flux analyzer (Seahorse Bioscience, Billerica, U.S.A.). PASMCs were seeded at a density of 1.0 × 10^4^/well in a Seahorse 96-well plate. A mitochondrial stress test was conducted using oligomycin (2.0 μM), carbonyl cyanide-p-trifluromethoxyphenyl-hydrazone (FCCP; 2.0 μM), and rotenone/antimycin A (0.5 μM). For the glycolysis stress test, glucose (10 mM), oligomycin (1.0 μM), and 2-deoxy-D-glucose (2-DG; 50 mM) were utilized. Data analysis was performed using Seahorse Wave software.

### Co-immunoprecipitation

Co-immunoprecipitation (Co-IP) was employed to investigate the interaction between two protein molecules. Utilizing the IP kit (P2179, Beyotime), primary PASMCs were collected and lysed according to the manufacturer’s instructions, yielding supernatants. These supernatants were then subjected to a BCA assay kit to determine the total protein concentration. Equal aliquots of the lysate supernatants were incubated overnight with 2 μg of either the target antibody or control IgG under constant rotation at 4°C. Following this, an A/G bead suspension was introduced and incubated for 2 hours at room temperature to facilitate the capture of immune complexes. The beads were subsequently washed with IP lysis buffer to remove unbound proteins. Finally, the samples were boiled in an SDS-PAGE loading buffer to prepare them for subsequent Western blot analysis.

### Glutathione S-transferase pull-down

The recombinant glutathione S-transferase (GST) and GST-LONP1 proteins were designed, synthesized, and purified by Gene Universal (Anhui, China). In this experiment, GST-LONP1 served as the bait protein, whereas MPC1 from cell lysates was the prey protein. The experiment adhered strictly to the manufacturer’s instructions provided with the GST pull-down kit (JKR23011, Genecreate, Wuhan, China). Briefly, 30 μl of magnetic beads was incubated with 200 μg of either GST or GST-LONP1 protein at room temperature for 2 hours, followed by washing. An equal volume of cell lysate supernatant was then added and incubated overnight at 4°C. The bound proteins were eluted using elution buffer, boiled, and subsequently analyzed by Western blot with target-specific antibodies.

### Cycloheximide chase assay

Cells undergoing various interventions were exposed to cycloheximide (CHX) (25 μg/ml; HY-12320, MedChemExpress) for durations of 0, 2, 6, and 10 hours. Following this, the cells were harvested and lysed to isolate proteins. Subsequently, Western blot was conducted to quantify the expression levels of the proteins at various time points, with the objective of assessing the half-life of MPC1.

### Molecular docking simulation

The three-dimensional structure of the target signaling protein, LONP1 (ID: 7KSL), was retrieved from the Research Collaboratory for Structural Bioinformatics Protein Data Bank website (https://www.rcsb.org/). Subsequently, the structure of the MPC1 protein (ID: AF-Q9Y5U8-F1-v4) was obtained from the AlphaFold Protein Structure Database website (https://alphafold.ebi.ac.uk/). To investigate the binding affinity and interaction pattern between LONP1 and MPC1 proteins, we utilized the online web server HADDOCK (https://wenmr.science.uu.nl/haddock2.4/) [25]. The cluster exhibiting the highest confidence score was selected as the optimal docking model and visualized through PyMol software (DeLano Scientific, San Carlos, U.S.A.).

### Statistical analysis

All statistical analyses were performed using GraphPad Prism software 9.1. Each experiment was independently replicated at least three times to ensure reproducibility. The data are presented as the mean ± standard deviation (SD). Student’s *t*-test was used for comparisons between two groups, while one or two-way analysis of variance (ANOVA) was employed for multiple group comparisons. Survival curves were generated using the Kaplan-Meier method and statistically compared using the log-rank test. Statistical significance was defined as a *P* value < 0.05.

## Results

### Up-regulated expression of LONP1 in PH

**Figure 1:**
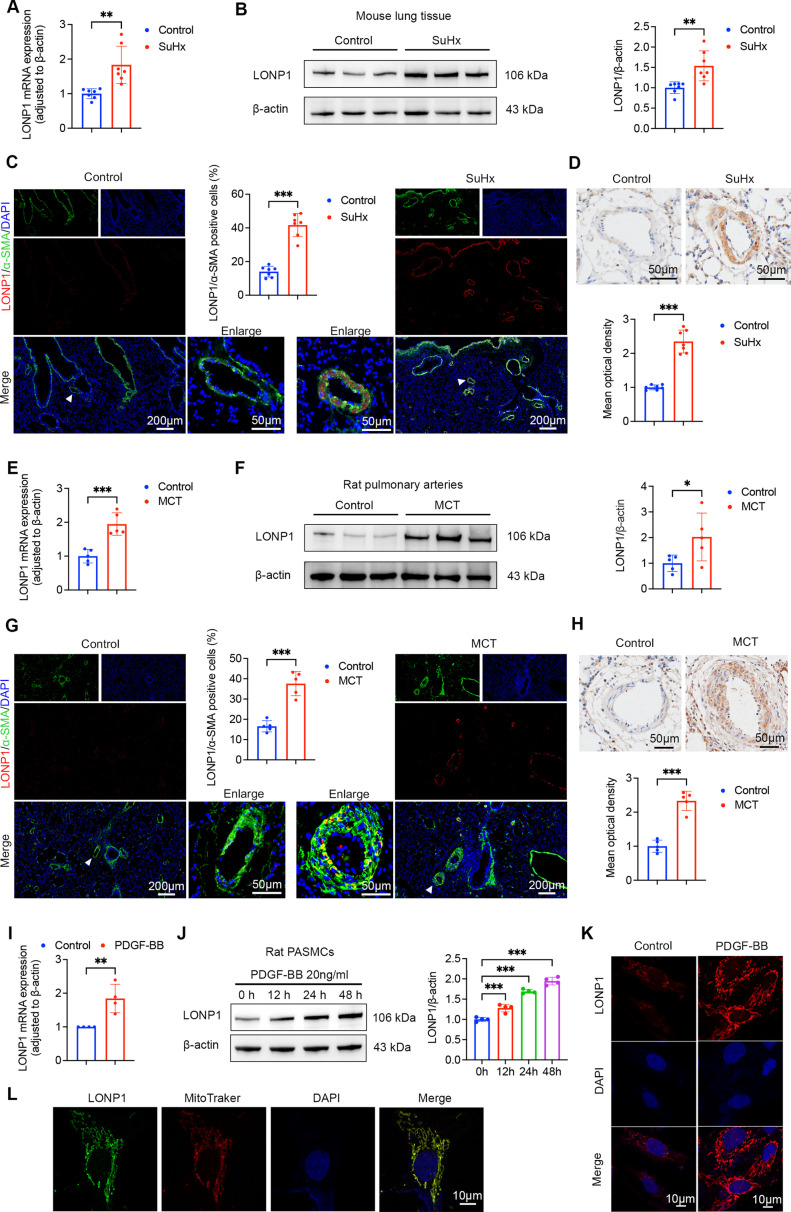
Up-regulated expression of LONP1 in PH. (**A**) qPCR analysis of LONP1 mRNA expression in lung tissue homogenates from control and SuHx-induced mice (*n* = 7). (**B**) Representative Western blot and quantitative analysis of LONP1 protein expression in lung tissue homogenates from control and SuHx-induced mice (*n* = 7). (**C**) Representative IF staining of LONP1 (red) and α-SMA (green) in lung sections from control and SuHx-induced mice, along with quantitative analysis (*n* = 7). Nuclei were stained with DAPI (blue). Scale bar = 200 µm and 50 µm. (**D**) Representative IHC staining of LONP1 in lung sections from control and SuHx-induced mice (*n* = 7). Scale bar = 50 µm. (**E**) qPCR analysis of LONP1 mRNA expression in pulmonary artery homogenates from control and MCT-induced rats (*n* = 5). (**F**) Representative Western blot and quantitative analysis of LONP1 protein expression in pulmonary artery homogenates from control and MCT-induced rats (*n* = 5). (**G**) Representative IF staining of LONP1 (red) and α-SMA (green) in lung sections from control and MCT-induced rats, along with quantitative analysis (*n* = 5). Nuclei were stained with DAPI (blue). Scale bar = 200 µm and 50 µm. (**H**) Representative IHC staining of LONP1 in lung sections from control and MCT-induced rats (*n* = 5). Scale bar = 50 µm. (**I**) qPCR analysis of LONP1 mRNA expression in PASMCs stimulated with 20 ng/ml PDGF-BB for 24 hours (*n* = 4). (**J**) Representative Western blot and quantitative analysis of LONP1 protein expression in PASMCs stimulated with 20 ng/ml PDGF-BB at the indicated time (*n* = 4). (**K**) Representative IF staining of LONP1 (red) in control and PDGF-BB-stimulated PASMCs. Nuclei were stained with DAPI (blue). Scale bar = 10 µm. (**L**) Representative IF staining of LONP1 (green) and MitoTracker (red) in PASMCs. Nuclei were stained with DAPI (blue). Scale bar = 10 µm. Data are presented as mean ± SD. Student’s *t*-test was used for comparisons between two groups, and one-way ANOVA with Bonferroni multiple comparisons test was used for comparisons among multiple groups. *P<0.05, **P<0.01, ***P<0.001. DAPI, 4’,6-diamidino-2-phenylindole; LONP1, Lon protease 1; PASMCs, pulmonary artery smooth muscle cells; PDGF-BB, platelet-derived growth factor BB; PH, pulmonary hypertension; qPCR, quantitative real-time polymerase chain reaction.

To investigate alterations in LONP1 expression during the pathogenesis of PH, we established two experimental PH models. The qPCR and Western blot analyses revealed a significant up-regulation of both mRNA and protein expression of LONP1 in the lung tissues of PH mice induced by SuHx (Figure 1A and B). IHC and IF staining further confirmed the expression of LONP1 in smooth muscle cells, fibroblasts, and endothelial cells. Notably, compared with the control group, the expression of LONP1 was exclusively elevated in PASMCs (α-SMA positive) of PH mice, with no significant differences observed in fibroblasts (vimentin positive) and endothelial cells [von Willebrand factor (vWF) positive] (Figure 1C and D and Supplementary Figure S1A and B). Similarly, pulmonary arteries isolated from MCT-induced PH rats exhibited significant increases in LONP1 mRNA and protein expression, primarily in PASMCs (Figure 1E–H and Supplementary Figure S1C and D). To further validate the expression in this cell type, primary PASMCs were successfully isolated and cultured from adult SD rats (Supplementary Figure S2). Platelet-derived growth factor-BB (PDGF-BB), a potent mitogen implicated in PH pathogenesis [26], stimulated a time- and concentration-dependent increase in LONP1 expression (Figure 1I–K and Supplementary Figure S3). Subsequently, IF staining was performed to assess the subcellular localization of LONP1 in PASMCs. The results indicated that LONP1 colocalized with MitoTracker, suggesting its enrichment primarily within the mitochondria of PASMCs (Figure 1L). Together, these findings suggest that increased expression of LONP1 in PASMCs may contribute to the development of PH.

### LONP1 knockdown prevents MCT-induced PH in rats

**Figure 2:**
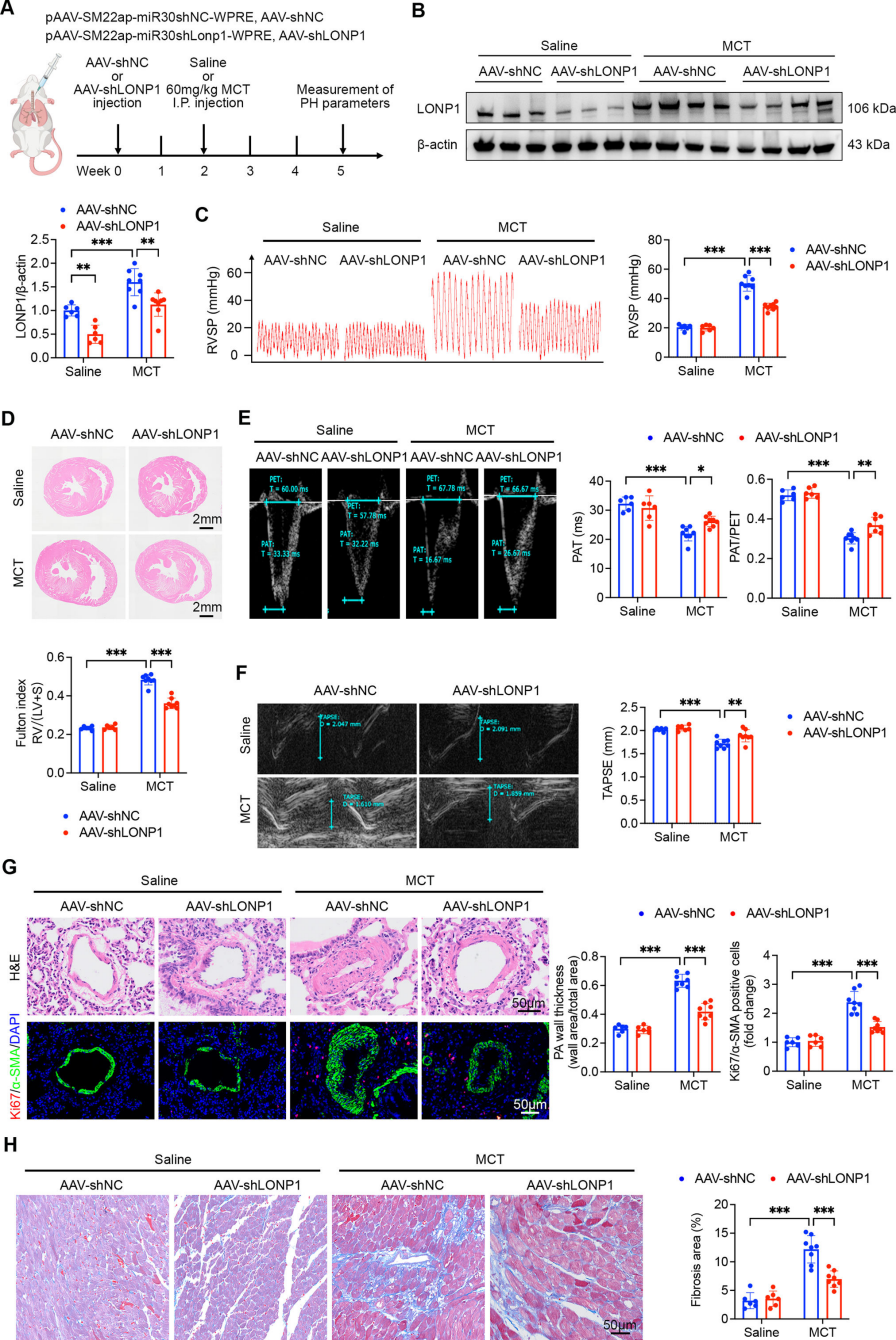
LONP1 knockdown prevents MCT-induced PH in rats. (**A**) Schematic illustration of the experimental design aimed at investigating the preventive effects of LONP1 knockdown on PH. (**B**) Representative Western blot and quantitative analysis of LONP1 protein expression in pulmonary artery homogenates from the AAV-shNC, AAV-shLONP1, AAV-shNC + MCT, and AAV-shLONP1 + MCT groups (*n* = 6–8). (**C**) Representative tracings and quantitative analysis of RVSP in each group (*n* = 6–8). (**D**) Representative H&E staining of heart sections and Fulton index in each group (*n* = 6–8). The Fulton index was calculated as the ratio of RV weight to the sum of LV and S weights. Scale bar = 2 mm. (**E** and **F**) Representative echocardiographic images and quantitative analysis of PAT, PAT/PET ratio, and TAPSE in each group (*n* = 6–8). (**G**) Representative H&E staining, Ki67 (red) and α-SMA (green) IF staining of lung tissue, and quantitative analysis of pulmonary artery wall thickness and Ki67/α-SMA positive cells in each group (*n* = 6–8). Nuclei were stained with DAPI (blue). Pulmonary artery wall thickness was calculated as the ratio of (total vascular area minus luminal area) to total vascular area. Scale bar = 50 µm. (**H**) Representative Masson’s trichrome stain images and quantitative analysis of RV fibrosis in each group (*n* = 6–8). Scale bar = 50 µm. Data are presented as mean ± SD. Two-way ANOVA with Bonferroni multiple comparisons test was used for comparisons among multiple groups. *P<0.05, **P<0.01, ***P<0.001. AAV, adeno-associated virus; ANOVA, analysis of variance; DAPI, 4’,6-diamidino-2-phenylindole; LONP1, Lon protease 1; LV, left ventricle; MCT, monocrotaline; PAT, pulmonary artery acceleration time; PET, pulmonary artery ejection time; PH, pulmonary hypertension; RV, right ventricle; RVSP, RV systolic pressure; TAPSE, tricuspid annular plane systolic excursion.

To investigate the involvement of LONP1 in the pathogenesis of PH *in vivo*, an AAV9 vector harboring SM22α promoter and shLONP1 sequence was employed to knock down LONP1 expression in PASMCs through intratracheal injection. Subsequently, we established an MCT-induced PH model and systematically evaluated PH-related physiological parameters in the rats five weeks post-AAV transfection. The experimental design is illustrated in Figure 2A. Western blot analysis and IF staining revealed a significant reduction in LONP1 protein expression in PASMCs following AAV-shLONP1 delivery, confirming the knockdown efficiency of AAV-shLONP1 (Figure 2B and Supplementary Figure S4). The results demonstrated that, compared with the AAV-shNC group, rats in the AAV-shNC + MCT group exhibited significantly elevated RVSP and Fulton index, which is a reliable indicator of RV hypertrophy. However, the increases in RVSP and Fulton index were significantly attenuated in the AAV-shLONP1 + MCT group compared with the AAV-shNC + MCT group (Figure 2C and D). Moreover, echocardiographic examination showed that PAT, PAT/PET ratio, and TAPSE were significantly decreased in the AAV-shNC + MCT group compared with the AAV-shNC group. In contrast, PAT, PAT/PET ratio, and TAPSE values were significantly increased in the AAV-shLONP1 + MCT group compared with the AAV-shNC + MCT group (Figure 2E and F). Consistent with the improvement in hemodynamic parameters, H&E staining of lung tissue demonstrated that LONP1 knockdown significantly alleviated MCT-induced pulmonary artery wall thickening (Figure 2G). Additionally, IF staining with Ki67 and α-SMA further confirmed the inhibitory effect of LONP1 knockdown on MCT-induced hyperproliferation of PASMCs in rats. Furthermore, we also found that the RV fibrosis in the AAV-shLONP1 + MCT group was significantly reduced compared with the AAV-shNC + MCT group (Figure 2H). Together, these findings indicate that knockdown of LONP1 effectively mitigates PVR and reduces pulmonary artery pressure as well as cardiac fibrosis.

### LONP1 promotes the proliferation and migration of PASMCs

**Figure 3:**
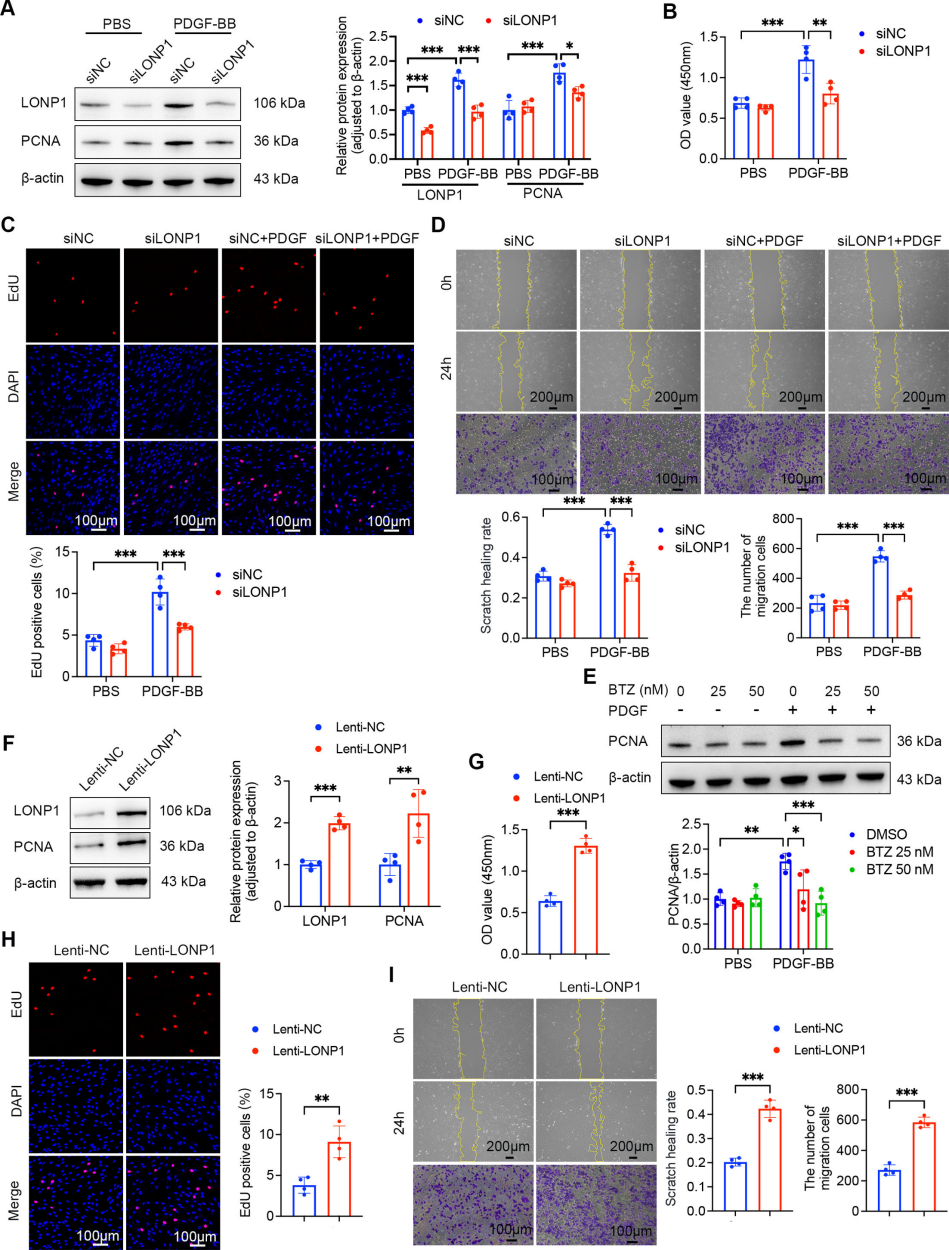
LONP1 promotes the proliferation and migration of PASMCs. (**A**) Representative Western blot and quantitative analysis of LONP1 and PCNA protein expression in PASMCs treated with siNC or siLONP1, with or without 20 ng/ml PDGF-BB stimulation for 24 hours (*n* = 4). (**B**) Cell viability was assessed by CCK-8 assay at 450 nm absorbance (*n* = 4). (**C**) Representative fluorescent images and quantitative analysis of EdU (red) incorporation into PASMCs (*n* = 4). Nuclei were stained with DAPI (blue). Scale bar = 100 µm. (**D**) Representative images and quantitative analysis of wound healing and Transwell assays assessing PASMC migration ability (*n* = 4). Scale bar = 200 µm for wound healing assay. Scale bar = 100 µm for Transwell assay. (**E**) Representative Western blot and quantitative analysis of PCNA protein expression in PASMCs treated with different concentrations of BTZ, with or without 20 ng/ml PDGF-BB stimulation for 24 hours (*n* = 4). (**F**) Representative Western blot and quantitative analysis of LONP1 and PCNA protein expression in PASMCs transfected with Lenti-NC or Lenti-LONP1 (*n* = 4). (**G**) Cell viability was assessed by CCK-8 assay at 450 nm absorbance (*n* = 4). (**H**) Representative fluorescent images and quantitative analysis of EdU (red) incorporation into PASMCs (*n* = 4). Nuclei were stained with DAPI (blue). Scale bar = 100 µm. (**I**) Representative images and quantitative analysis of wound healing and Transwell assays (*n* = 4). Scale bar = 200 µm for wound healing assay. Scale bar = 100 µm for Transwell assay. Data are presented as mean ± SD. Student’s t-test was used for comparisons between two groups, and two-way ANOVA with Bonferroni multiple comparisons test was used for comparisons among multiple groups. *P<0.05, **P<0.01, ***P<0.001. DAPI, 4’,6-diamidino-2-phenylindole; LONP1, Lon protease 1; PASMCs, pulmonary artery smooth muscle cells; PCNA, proliferating cell nuclear antigen.

The excessive proliferation and migration of PASMCs are recognized as critical factors in PVR associated with PH [27]. Considering the *in vivo* role of LONP1, primary rat PASMCs were isolated and cultured to elucidate the specific effects of LONP1 on their proliferation and migration *in vitro*. We designed and screened siRNAs effectively targeting LONP1. qPCR and Western blot analyses revealed significant reductions in both mRNA and protein expression of LONP1 in PASMCs after siRNA transfection (Supplementary Figure S5). Based on the knockdown efficiency, siLONP1-1 was chosen for subsequent experiments. As depicted in Figure 3A, knockdown of LONP1 markedly inhibited the PDGF-BB-induced up-regulation of PCNA, a cell proliferation marker. Consistent with this finding, CCK-8 and EdU staining assays further confirmed the inhibitory effect of LONP1 knockdown on PDGF-BB-induced proliferation of PASMCs *in vitro* (Figure 3B and C). Additionally, wound healing and Transwell migration assays demonstrated that PDGF-BB induced migration of PASMCs, which was significantly suppressed by LONP1 knockdown (Figure 3D). BTZ, a clinically used proteasome inhibitor for multiple myeloma treatment, has been reported to inhibit LONP1 protease activity [28–30]. Hence, we treated PASMCs with various concentrations of BTZ to determine the optimal concentration. Western blot analysis indicated that BTZ inhibited PDGF-BB-induced PCNA expression in a concentration-dependent manner (Figure 3E). To minimize toxicity to PASMCs, 25 nM BTZ was selected for subsequent experiments. Similar to the effects observed with siLONP1, BTZ significantly inhibited the increased cell viability and migration capacity of PASMCs under PDGF-BB treatment (Supplementary Figure S6). To further validate the functional role of LONP1, a lentivirus vector overexpressing LONP1 was constructed and transfected into PASMCs (Supplementary Figure S7). Experimental results indicated that LONP1 overexpression significantly increased PCNA expression, cell viability, and EdU-positive cell ratio in PASMCs, as well as enhanced their migration capacity (Figure 3F–I). Together, these findings suggest that LONP1 is both necessary and sufficient for promoting the proliferation and migration of PASMCs.

### LONP1 promotes glycolytic reprogramming

**Figure 4:**
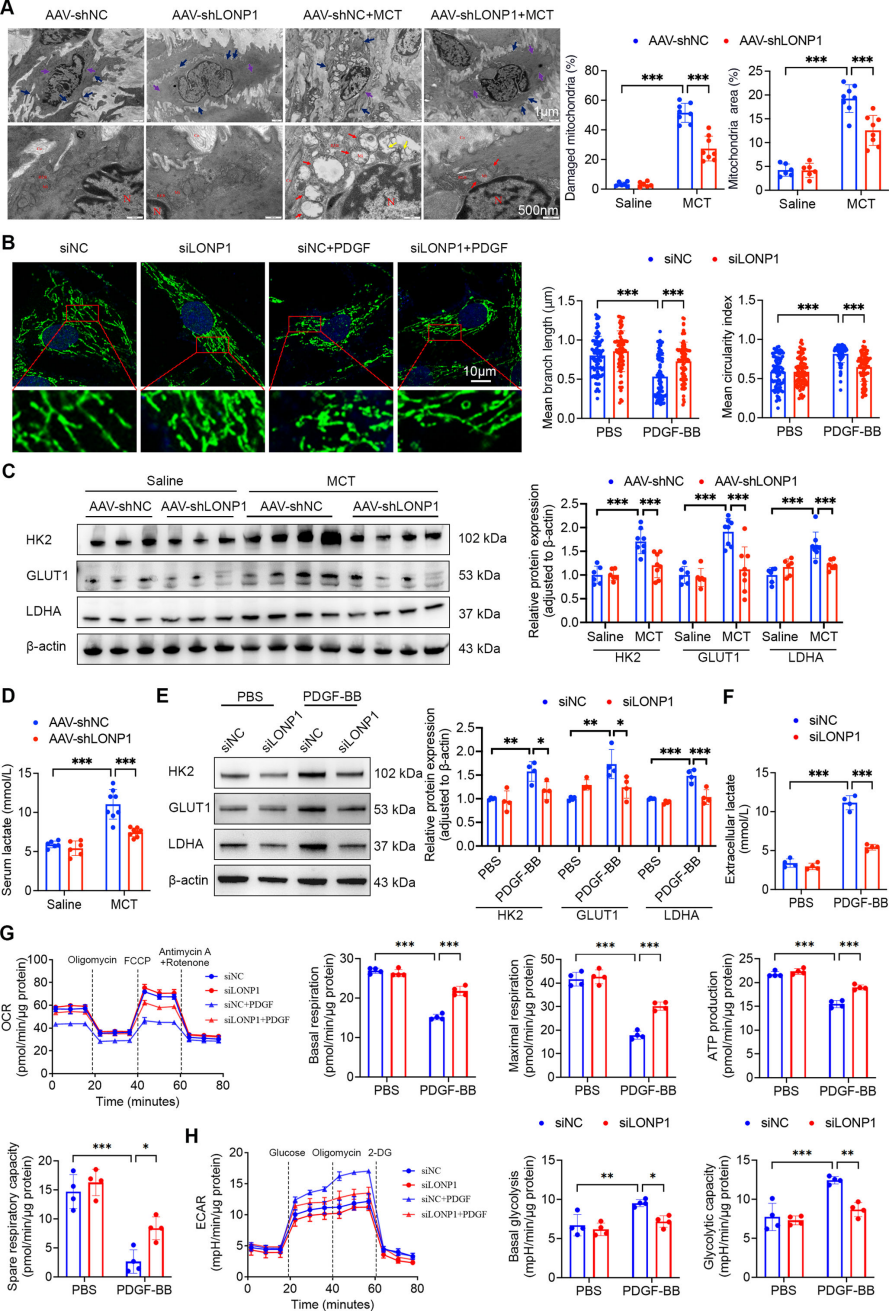
LONP1 facilitates glycolytic reprogramming. (**A**) Representative TEM images of mitochondria in PASMCs from pulmonary arteries in each group, along with quantitative analysis (*n* = 6–8, 20 PASMCs/independent experiment). Dense body and dense patch are specific structures of smooth muscle cells. Dense body, dense patch, and damaged mitochondria are indicated with purple, dark blue, and red arrows, respectively. Scale bar = 1 μm and 500 nm. (**B**) Representative IF staining of MitoTracker (green) in PASMCs from the siNC, siLONP1, siNC+PDGF-BB, and siLONP1 + PDGF-BB groups, along with quantitative analysis (*n* = 4, 20 PASMCs/independent experiment). Nuclei were stained with Hoechst 33,342 (blue). Scale bar = 10 µm. (**C**) Representative Western blot and quantitative analysis of HK2, GLUT1, and LDHA protein expression in pulmonary artery homogenates from each group (*n* = 6–8). (**D**) Serum lactate levels from each group (*n* = 6–8). (**E**) Representative Western blot and quantitative analysis of HK2, GLUT1, and LDHA protein expression in PASMCs from each group (*n* = 4). (**F**) Lactate levels in the supernatants of PASMC cultures from each group (*n* = 4). (**G**) Real-time monitoring of the OCR in PASMCs with the indicated treatments, along with quantitative analysis (*n* = 4). (**H**) Real-time monitoring of the ECAR in PASMCs with the indicated treatments, along with quantitative analysis (*n* = 4). Data are presented as mean ± SD. Two-way ANOVA with Bonferroni multiple comparisons test was used for comparisons among multiple groups. *P<0.05, **P<0.01, ***P<0.001. LONP1, Lon protease 1; PASMCs, pulmonary artery smooth muscle cells; TEM, transmission electron microscopy.

Glycolytic reprogramming is a fundamental mechanism driving the hyperproliferation of PASMCs during the progression of PH [31]. To investigate the potential role of LONP1 in modulating glycolytic reprogramming in PH, we initially assessed ATP levels in the pulmonary arteries. Compared with the AAV-shNC group, ATP levels were significantly decreased in the AAV-shNC + MCT group. Conversely, ATP levels rebounded in the AAV-shLONP1 + MCT group relative to the AAV-shNC + MCT group (Supplementary Figure S8A). To further elucidate the determinants of ATP levels, we prepared samples using semi-thin and ultra-thin sectioning techniques and examined the mitochondrial morphology of PASMCs via TEM. Significant alterations in mitochondrial morphology, including swelling, cristae disruption, matrix depletion, vacuolization, and outer membrane rupture, were observed in the AAV-shNC + MCT group compared with the AAV-shNC group. However, the administration of AAV-shLONP1 markedly alleviated these MCT-induced mitochondrial abnormalities (Figure 4A). *In vitro*, PDGF-BB caused the mitochondria of PASMCs to transition from elongated tubular structures to fragmented spherical morphologies; however, knockdown of LONP1 significantly inhibited this morphological transformation (Figure 4B). The glycolysis was subsequently analyzed. LONP1 knockdown significantly reduced the elevated protein levels of GLUT1, HK2, and LDHA in MCT-induced rats (Figure 4C). Moreover, LONP1 knockdown also inhibited the MCT-induced accumulation of serum lactate (Figure 4D). Consistently, PDGF-BB induced decreases in ATP levels; increases in GLUT1, HK2, and LDHA protein expression; and extracellular lactate levels in PASMCs, whereas LONP1 knockdown *in vitro* significantly reversed these effects (Figure 4E and F and Supplementary Figure S8B). Conversely, LONP1 overexpression led to opposing results (Supplementary Figure S9). To comprehensively investigate the precise impact of LONP1 on cellular energy metabolism, the XF-96 extracellular flux analyzer was employed to perform mitochondrial stress tests and glycolysis stress tests, accurately measuring the OCR and ECAR of PASMCs. The results revealed that PDGF-BB treatment had a significant inhibitory effect on the mitochondrial energy metabolism of PASMCs, as evidenced by substantial decreases in basal respiration, maximal respiration, ATP production, and spare respiratory capacity (Figure 4G). Furthermore, PDGF-BB treatment notably augmented both basal glycolysis and glycolytic capacity (Figure 4H). Importantly, knockdown of LONP1 significantly reversed the mitochondrial metabolic dysfunction and the abnormal up-regulation of glycolysis induced by PDGF-BB. Therefore, these data suggest that LONP1 promotes mitochondrial damage and metabolic shift toward glycolysis in PASMCs.

### MPC1 is a potential downstream target of LONP1 and regulates PASMC phenotype

**Figure 5:**
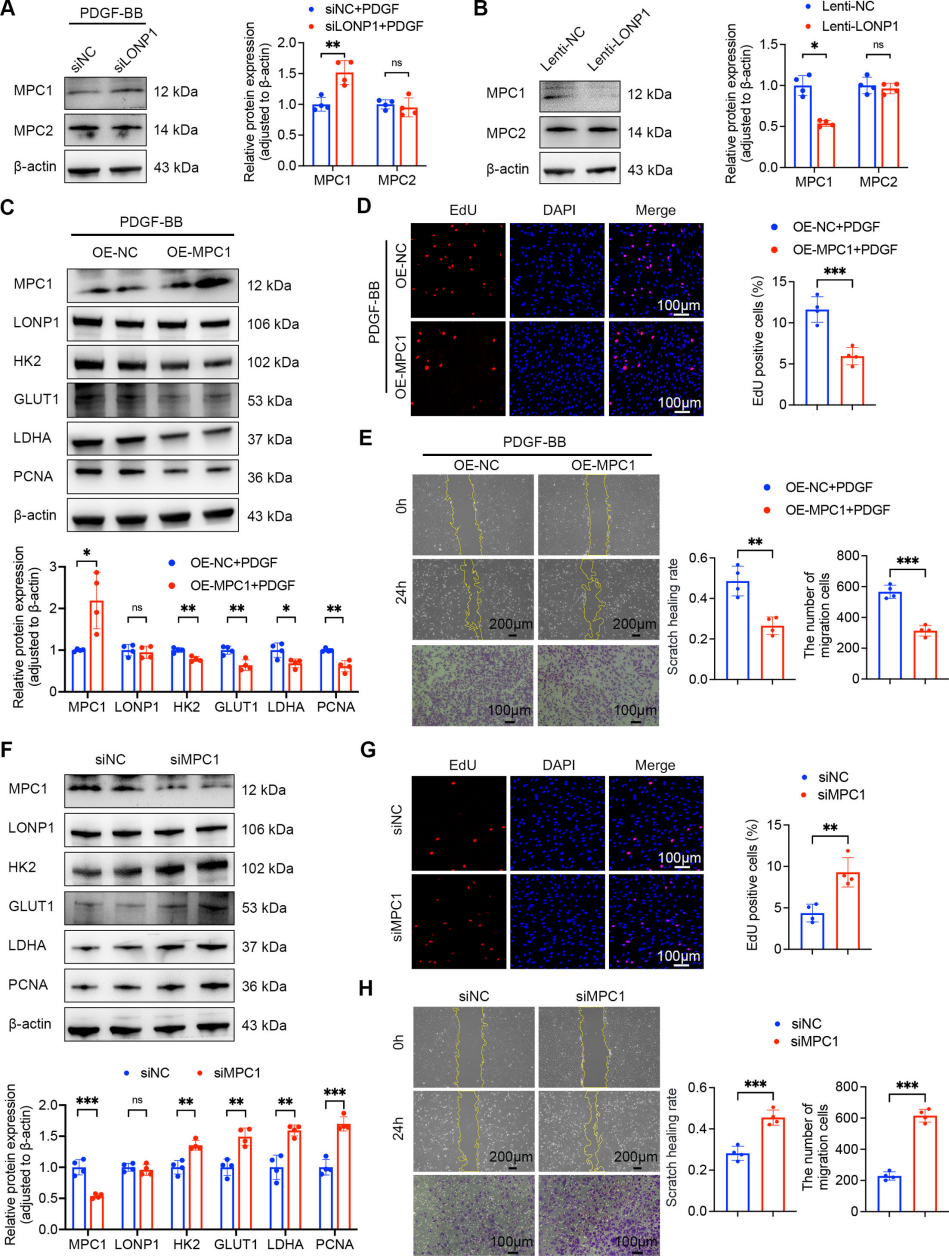
MPC1 is a potential downstream target of LONP1 and regulates PASMC phenotype. (**A**) Representative Western blot and quantitative analysis of MPC1 and MPC2 protein expression in PASMCs treated with PDGF-BB and transfected with either siNC or siLONP1 (*n* = 4). (**B**) Representative Western blot and quantitative analysis of MPC1 and MPC2 protein expression in PASMCs transfected with either Lenti-NC or Lenti-LONP1 (*n* = 4). (**C**) Representative Western blot and quantitative analysis of MPC1, LONP1, HK2, GLUT1, LDHA, and PCNA protein expression in PASMCs treated with PDGF-BB and transfected with either OE-NC or OE-MPC1 plasmids (*n* = 4). (**D**) Representative fluorescent images and quantitative analysis of EdU (red) incorporation into PASMCs (*n* = 4). Nuclei were stained with DAPI (blue). Scale bar = 100 µm. (**E**) Representative images and quantitative analysis of wound healing and Transwell assays (*n* = 4). Scale bar = 200 µm for wound healing assay. Scale bar = 100 µm for Transwell assay. (**F**) Representative Western blot and quantitative analysis of MPC1, LONP1, HK2, GLUT1, LDHA, and PCNA protein expression in PASMCs transfected with either siNC or siMPC1 (*n* = 4). (**G**) Representative fluorescent images and quantitative analysis of EdU (red) incorporation into PASMCs (*n* = 4). Nuclei were stained with DAPI (blue). Scale bar = 100 µm. (**H**) Representative images and quantitative analysis of wound healing and Transwell assays (*n* = 4). Scale bar = 200 µm for wound healing assay. Scale bar = 100 µm for Transwell assay. Data are presented as mean ± SD. Student’s *t*-test was used for comparisons between two groups. *P<0.05, **P<0.01, ***P<0.001. DAPI, 4’,6-diamidino-2-phenylindole; GLUT1, glucose transporter-1; LDHA, lactate dehydrogenase A; LONP1, Lon protease 1; MPC1, mitochondrial pyruvate carrier 1; PASMCs, pulmonary artery smooth muscle cells; PCNA, proliferating cell nuclear antigen.

MPC complex, composed of MPC1 and MPC2 subunits, constitutes the sole identified pathway for the translocation of pyruvate from the cytosol to mitochondria [32]. A study conducted by Pollecker et al. [33] demonstrated that silencing of LONP1 resulted in a notable alteration in the overall abundance of mitochondrial proteins, with a striking elevation in MPC protein expression, exceeding a 20-fold increase. This intriguing finding prompted us to delve deeper into the specific role of MPC in LONP1-mediated glycolytic reprogramming. qPCR and Western blot were thus employed to assess the expression of MPC1 and MPC2 in PASMCs under conditions of LONP1 knockdown and overexpression. In comparison with the control group, the expression of MPC1 protein was notably elevated in PASMCs with LONP1 knockdown, whereas it was markedly decreased in PASMCs with LONP1 overexpression. Conversely, the expression of MPC2 remained consistent across all groups (Figure 5A and B). Notably, MPC1 mRNA expression remained consistent regardless of LONP1 knockdown or overexpression (Supplementary Figure S10). These findings suggest that MPC1 may serve as a potential downstream target of LONP1. To further dissect the functional implications of MPC1, plasmids for MPC1 overexpression and siRNAs for MPC1 knockdown were constructed and successfully transfected into PASMCs (Supplementary Figure S11). As illustrated in Figure 5C, under PDGF-BB stimulation, the expression of GLUT1, HK2, LDHA, and PCNA proteins was markedly suppressed in PASMCs overexpressing MPC1. Correspondingly, the results of EdU staining, wound healing, and Transwell assays provided additional evidence of the significant inhibitory effect of MPC1 overexpression on PDGF-BB-induced proliferation and migration of PASMCs (Figure 5D and E). Conversely, MPC1 knockdown led to a significant up-regulation of GLUT1, HK2, LDHA, and PCNA proteins in PASMCs, with the results of EdU staining, wound healing, and Transwell assays confirming that MPC1 knockdown significantly promoted the proliferation and migration of PASMCs (Figure 5F–H). Together, these findings underscore the pivotal role of MPC1 in regulating glycolytic reprogramming, proliferation, and migration of PASMCs.

### LONP1 facilitates the glycolytic reprogramming by degrading MPC1, thereby promoting cellular proliferation and migration

**Figure 6:**
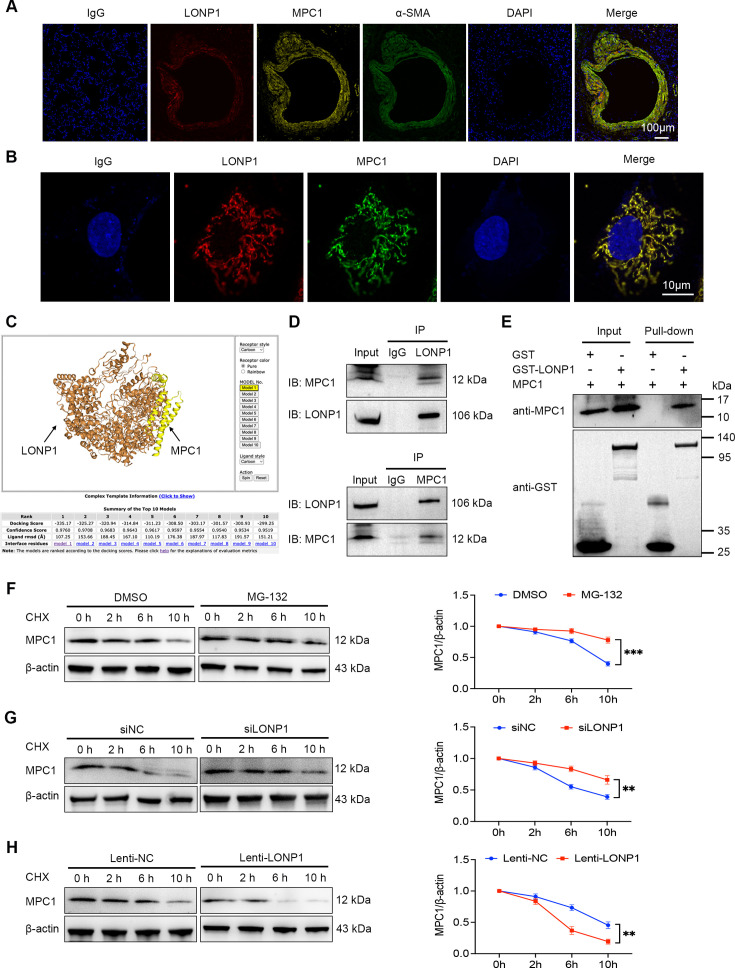
MPC1 functions as a degradation substrate for LONP1. (**A**) Representative IF staining of LONP1 (red), MPC1 (yellow), and α-SMA (green) in rat lung sections. IgG was used for negative control staining. Nuclei were stained with DAPI (blue). Scale bar = 100 µm. (**B**) Representative IF staining of LONP1 (red) and MPC1 (green) in PASMCs. IgG was used for negative control staining. Nuclei were stained with DAPI (blue). Scale bar = 10 µm. (**C**) Simulated docking of LONP1 and MPC1 protein molecules using the protein-protein interaction prediction software HADDOCK. (**D**) Co-IP using either LONP1 or MPC1 antibodies confirmed the interaction between LONP1 and MPC1 in PASMCs (*n* = 4). (**E**) GST pull-down was conducted to detect the direct interaction between LONP1 and MPC1 (*n* = 4). Purified GST or GST-LONP1 was mixed with PASMC lysates, followed by precipitation using glutathione magnetic beads. (**F-H**) Representative Western blot and quantitative analysis of MPC1 protein expression in PASMCs (*n* = 4). PASMCs were treated with CHX (25 µg/ml) for 0, 2, 6, and 10 hours after intervention with MG-132 (5 μM) (**F**), transfection with siNC or siLONP1 (**G**), or transfection with Lenti-NC or Lenti-LONP1 (**H**). Cell samples were collected and lysed for Western blot to detect MPC1 expression. Data are presented as mean ± SD. Student’s *t*-test was used for comparisons between two groups. *P<0.05, **P<0.01, ***P<0.001. DAPI, 4’,6-diamidino-2-phenylindole; GLUT1, glucose transporter-1; LDHA, lactate dehydrogenase A; LONP1, Lon protease 1; MPC1, mitochondrial pyruvate carrier 1; PASMCs, pulmonary artery smooth muscle cells; PCNA, proliferating cell nuclear antigen.

To gain further insights into the mechanisms underlying LONP1-mediated regulation of MPC1 expression, we initiated an investigation of the localization relationship between LONP1 and MPC1, both *in vivo* and *in vitro*. IF staining revealed significant co-localization of LONP1 and MPC1 in pulmonary arteries and PASMCs (Figure 6A and B). Next, the protein–protein interaction prediction tool HADDOCK was utilized to identify multiple high-confidence interaction patterns between LONP1 and MPC1 (Figure 6C). Moreover, Co-IP analysis indicated that endogenous LONP1 interacted with and precipitated MPC1 in PASMCs. Conversely, endogenous MPC1 also interacted with and precipitated LONP1 (Figure 6D). Furthermore, pull-down assays employing recombinant GST-fused proteins confirmed that GST-LONP1 could directly bind to and capture MPC1 *in vitro*, whereas the GST control failed to exhibit such binding (Figure 6E). Considering the proteasome property of LONP1, we thus employed CHX to inhibit protein synthesis and assessed the impact of LONP1 on the degradation rate of MPC1. The proteasome inhibitor MG-132 significantly inhibited the degradation of MPC1, thereby extending its half-life (Figure 6F). Consistent with this, MPC1 exhibited a prolonged half-life in PASMCs with LONP1 knockdown (Figure 6G). Conversely, MPC1 underwent rapid degradation in PASMCs overexpressing LONP1 (Figure 6H).

**Figure 7:**
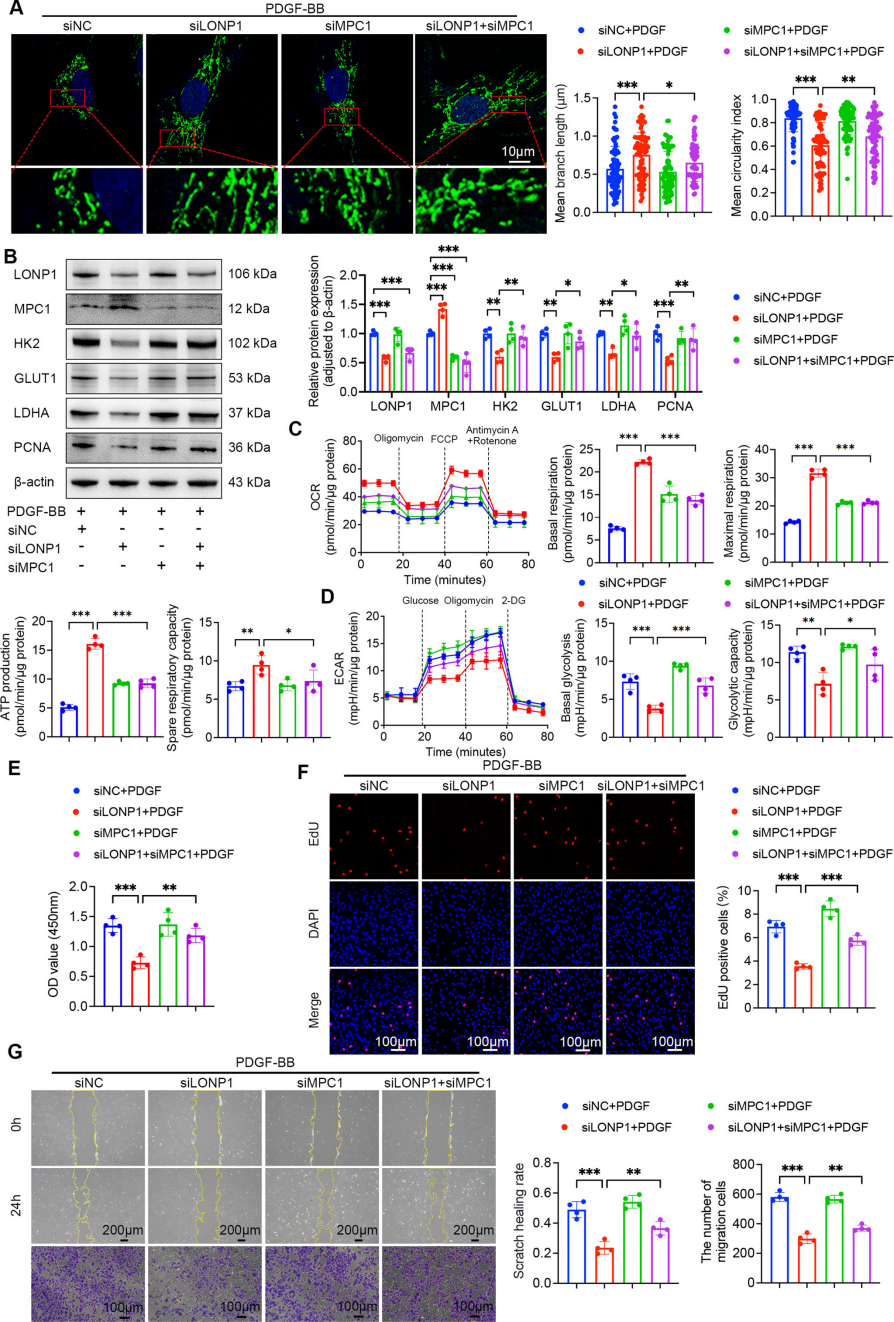
MPC1 knockdown abrogates the suppressive effects of LONP1 knockdown on glycolytic reprogramming, proliferation, and migration in PASMCs. (**A**) Representative IF and quantitative analysis of mitochondria stained with MitoTracker (green) in PASMCs (*n* = 4). Nuclei were stained with Hoechst 33,342 (blue). Scale bar = 10 µm. (**B**) Representative Western blot and quantitative analysis of LONP1, MPC1, HK2, GLUT1, LDHA, and PCNA protein expression in PASMCs (*n* = 4). (**C**) Real-time monitoring of the OCR in PASMCs with the indicated treatments, along with quantitative analysis (*n* = 4). (**D**) Real-time monitoring of the ECAR in PASMCs with the indicated treatments, along with quantitative analysis (*n* = 4). (**E**) Cell viability was assessed by CCK-8 assay at 450 nm absorbance (*n* = 4). (**F**) Representative fluorescent images and quantitative analysis of EdU (red) incorporation in PASMCs (*n* = 4). Nuclei were stained with DAPI (blue). Scale bar = 100 µm. (**G**) Representative images and quantitative analysis of wound healing and Transwell assays in PASMCs (*n* = 4). Scale bar = 200 µm for wound healing assay. Scale bar = 100 µm for Transwell assay. Data are presented as mean ± SD. One-way ANOVA with Bonferroni multiple comparisons test was used for comparisons among multiple groups. *P<0.05, **P<0.01, ***P<0.001. LONP1, Lon protease 1; MPC1, mitochondrial pyruvate carrier 1; PASMCs, pulmonary artery smooth muscle cells.

Having identified MPC1 as a degradation substrate of LONP1, we subsequently employed siRNAs to knockdown both LONP1 and MPC1, aiming to elucidate the role of LONP1-mediated MPC1 degradation in glycolytic reprogramming, proliferation, and migration of PASMCs. The results demonstrated that the rescue effect of LONP1 knockdown on the reduction in ATP levels in PDGF-BB-treated PASMCs was inhibited by MPC1 knockdown (Supplementary Figure S12). Moreover, LONP1 knockdown significantly mitigated PDGF-BB-induced mitochondrial damage and up-regulation of key glycolytic enzymes. However, this protective effect was markedly abolished by MPC1 knockdown (Figure 7A and B). The knockdown of MPC1 also eliminated the inhibitory effect of LONP1 knockdown on PDGF-BB-induced mitochondrial metabolic disruption and enhanced glycolysis (Figure 7C and D). Consistently, LONP1 knockdown significantly suppressed PDGF-BB-induced proliferation and migration, whereas MPC1 knockdown reversed this suppression (Figure 7E–G). Together, these findings support a model in which LONP1 directly interacts with and degrades MPC1, thereby facilitating glycolytic reprogramming and promoting the proliferation and migration of PASMCs.

### Knockdown or pharmacological inhibition of LONP1 reverses MCT-induced PH in rats

**Figure 8:**
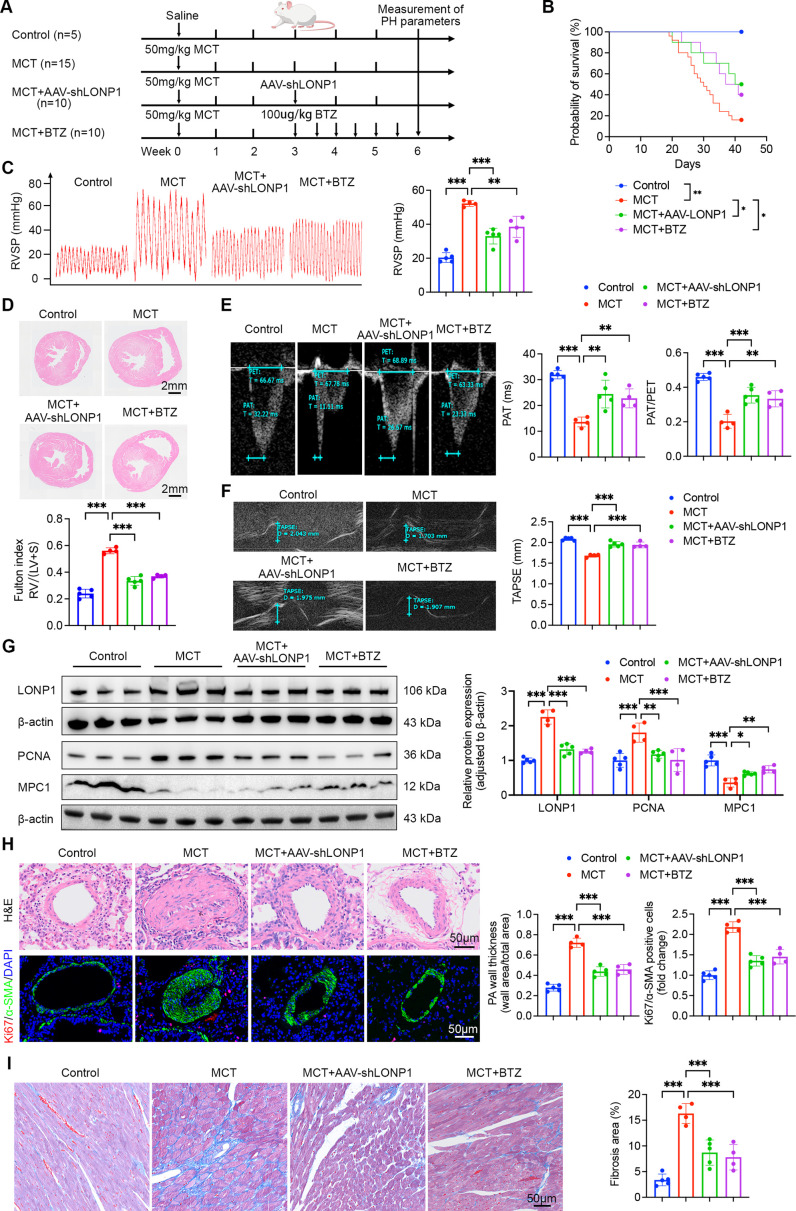
Knockdown or pharmacological inhibition of LONP1 reverses MCT-induced PH in rats. (**A**) Schematic illustration of the experimental design aimed at investigating the therapeutic effects of LONP1 knockdown or pharmacological inhibition on PH. (**B**) Kaplan–Meier survival curves for rats in the control, MCT, MCT + AAV-shLONP1, and MCT + BTZ groups (*n* = 5–15). (**C**) Representative tracings and quantitative analysis of RVSP in each group (*n* = 4–5). (**D**) Representative H&E staining of heart sections and Fulton index in each group (*n* = 4–5). The Fulton index was calculated as the ratio of RV weight to the sum of LV and S weights. Scale bar = 2 mm. (**E** and **F**) Representative echocardiographic images and quantitative analysis of PAT, PAT/PET ratio, and TAPSE in each group (*n* = 4–5). (**G**) Representative Western blot and quantitative analysis of LONP1, PCNA, and MPC1 protein expression in pulmonary artery homogenates from each group (*n* = 4–5). (**H**) Representative H&E staining, Ki67 (red) and α-SMA (green) IF staining of lung tissue, and quantitative analysis of pulmonary artery wall thickness and Ki67/α-SMA positive cells in each group (*n* = 4–5). Nuclei were stained with DAPI (blue). Pulmonary artery wall thickness was calculated as the ratio of (total vascular area minus luminal area) to total vascular area. Scale bar = 50 µm. (**I**) Representative Masson’s trichrome stain images and quantitative analysis of RV fibrosis in each group (*n* = 4–5). Scale bar = 50 µm. Data are presented as mean ± SD. Log-rank test and one-way ANOVA with Bonferroni multiple comparisons test were used for comparisons among multiple groups. *P<0.05, **P<0.01, ***P<0.001. AAV, adeno-associated virus; H&E hematoxylin and eosin; LONP1, Lon protease 1; MCT, monocrotaline; PCNA, proliferating cell nuclear antigen; PH, pulmonary hypertension.

To evaluate the therapeutic potential of targeting LONP1, we conducted a comprehensive investigation utilizing a rat model of MCT-induced PH. Rats were administered either AAV-shLONP1 or BTZ. The experimental design is illustrated in Figure 8A. Both AAV-shLONP1 and BTZ treatments significantly prolonged the survival duration of PH rats (Figure 8B). Moreover, compared with the control group, rats treated with either AAV-shLONP1 or BTZ exhibited a notable decrease in RVSP and Fulton index (Figure 8C and D). Echocardiographic examination also revealed improvements in crucial parameters, such as PAT, PAT/PET ratio, and TAPSE in PH rats treated with AAV-shLONP1 or BTZ (Figure 8E and F). Western blot analysis showed a significant decrease in PCNA expression in rats treated with AAV-shLONP1 or BTZ, suggesting a suppression of cellular proliferation (Figure 8G). Lung histopathology further corroborated these findings, demonstrating a substantial reduction in MCT-induced pulmonary artery wall thickening and proliferation of PASMCs (Figure 8H). Moreover, the level of RV fibrosis in rats treated with AAV-shLONP1 or BTZ was significantly reduced (Figure 8I). These results provide compelling evidence for the potential of LONP1 knockdown or pharmacological inhibition as a novel therapeutic approach for PH treatment.

## Discussion

In the pulmonary vascular system, the excessive proliferation and migration of PASMCs, coupled with their resistance to apoptosis, are intimately correlated with the severity of PVR and pulmonary artery pressure [34–36]. Consequently, the effective attenuation of PASMC proliferation and migration is crucial for halting the pathological progression of PH. In the present study, we provide novel evidence indicating that LONP1 facilitates glycolytic reprogramming, promoting abnormal cell proliferation and migration, ultimately contributing to PVR. This effect is precipitated through the degradation of MPC1. The knockdown or pharmacological inhibition of LONP1 attenuated PASMC proliferation and migration, thereby exerting both preventive and therapeutic benefits in PH.

Our study elucidates the pivotal role of LONP1 in the pathogenesis of PH. LONP1 is a prominent and crucial AAA^+^ protease located in mitochondria, functioning in a soluble hexameric ring configuration to bind and degrade a range of substrates [37]. It plays a fundamental role in maintaining mitochondrial homeostasis. Prior research has demonstrated that elevated expression of LONP1 in cancer enables cancer cells to resist oncogenic stressors, including hypoxia, protein damage, and oxidative stress, and alters energy metabolic pathways, thereby fostering malignant proliferation, tumor metastasis, and therapeutic resistance [19,38–40]. Powering down LONP1 has currently emerged as a new strategy for cancer treatment. Consistent with these cancer-related findings, we observed an up-regulation of LONP1 expression in lung tissues from two PH models and in PDGF-BB-induced PASMCs. Gain- and loss-of-function experiments confirmed that LONP1 promoted the proliferation and migration of PASMCs. By employing AAV-shLONP1 for LONP1 knockdown *in vivo*, a significant alleviation was observed in the counts of Ki67/α-SMA-positive cells, pulmonary artery wall thickness, Fulton index, and RVSP. In established PH models, treatment with AAV-shLONP1 or BTZ markedly reversed PASMC hyperproliferation, PVR, and RV hypertrophy. These data strongly support the contention that up-regulated LONP1 in PASMCs mediates the PVR process in PH.

Glycolytic reprogramming is a fundamental mechanism underlying the onset and progression of PH [41]. In PH, there is a metabolic shift in glucose metabolism, transitioning from OXPHOS to glycolysis. This shift not only ensures adequate cellular carbon sources but also augments cell proliferation through paracrine and autocrine signaling mediated by local metabolites [42,43]. Our study revealed that in pulmonary artery homogenates of MCT-induced PH rats, ATP levels in PASMCs decreased, mitochondria were severely compromised, and the expression of GLUT1 and key glycolytic enzymes increased. Additionally, there was an excessive accumulation of serum lactate. Notably, the proliferation of PASMCs in the lung tissues of MCT-induced rats was significantly elevated compared to the control group. These findings further underscore the significance of glycolytic reprogramming in PH pathogenesis. Numerous studies have demonstrated that targeting various key glycolytic enzymes, such as HK2, LDHA, and 6-phosphofructo-2-kinase/fructose-2,6-bisphosphatase 3, can effectively reverse metabolic shifts and cellular hyperproliferation [9,11,44]. Thus, inhibiting the glycolytic pathway emerges as a potential therapeutic strategy for PH.

Previous research has emphasized LONP1 as a key protein regulating glycolytic reprogramming, influencing cell proliferation and apoptosis. In melanoma cells, LONP1 overexpression disrupted the assembly and function of ETC complex I, leading to the down-regulation of mitochondrial respiration and up-regulation of the glycolytic pathway, which promoted cell proliferation and invasion [15]. In cardiomyocytes, LONP1 overexpression mediated glycolytic reprogramming by degrading pyruvate dehydrogenase (PDH) kinase 4 or reducing the expression and activity of ETC complex I, thereby decreasing cell apoptosis and exerting cardioprotective effects [45,46]. In primary fibroblasts, LONP1 silencing inhibited PDH and aconitase activity by reducing the degradation of phosphorylated E1α and oxidized aconitase, resulting in a metabolic shift toward glycolysis and cell apoptosis [47,48]. Here, we established a novel connection between external pathogenic factors (such as hypoxia and PDGF-BB) and metabolic shifts in PASMCs through LONP1. We found that LONP1 knockdown, both *in vitro* and *in vivo*, significantly ameliorated mitochondrial damage, suppressed glycolysis, and curbed proliferation of PASMCs.

To elucidate the mechanism by which LONP1 participates in glycolytic reprogramming in PH, qPCR and Western blot analyses were conducted based on prior proteomic findings. These analyses suggested that LONP1 might regulate the protein expression of MPC1. Further assays confirmed that MPC1 was a substrate directly bound and degraded by LONP1. MPC, a heterodimer composed of MPC1 and MPC2 subunits, is located in the mitochondrial inner membrane and ubiquitously expressed in all human tissues [49]. Initially identified in 2012, MPC transports pyruvate produced by cytosolic glycolysis into the mitochondrial matrix, thereby providing the primary substrate for the tricarboxylic acid cycle and subsequent OXPHOS [50]. As a pivotal node linking glycolysis and mitochondrial metabolism, the abundance and activity of MPC proteins determine the metabolic fate of pyruvate, directing it toward either mitochondrial OXPHOS or cytosolic glycolysis [32]. Studies have demonstrated that MPC expression is decreased in pancreatic cancer, colorectal cancer, and glioblastoma, and MPC expression is negatively correlated with poor prognosis [51–53]. Specifically, MPC1 knockdown has been shown to increase glycolytic levels and cell invasion in tumor cells, highlighting its pivotal role in regulating tumor cell growth [54,55]. Additionally, loss-of-function mutations of MPC1 or MPC2 prevent MPC assembly, and mitochondrial pyruvate uptake, leading to significant metabolic acidosis and hyperlactatemia [56,57]. We have previously reported that MPC1 knockdown or pharmacological inhibition in vascular endothelial cells results in increased glycolytic enzyme expression and enhanced lactate secretion [58]. In the present study, MPC1 knockdown significantly promoted glycolysis, proliferation, and migration of PASMCs, whereas MPC1 overexpression had the opposite effects. Although some studies have reported that the loss of one MPC subunit leads to the degradation of the other subunit [59,60], our data indicated that LONP1 knockdown or overexpression affected MPC1 but not MPC2 protein expression. Similarly, Manzila et al. [53] reported unchanged mRNA and protein expression of MPC1 in colorectal tumor cells with MPC2 knockdown or overexpression. This finding may be attributed to specific regulatory mechanisms in different cell types and pathological states.

The limitations of this study encompass several aspects. First, the absence of a conditional knockout mouse model specifically targeting LONP1 in smooth muscle cells represents a shortcoming in our investigation. Nevertheless, the remarkable therapeutic effect observed with *in vivo* LONP1 knockdown using AAV vectors still holds promise for clinical translation. Second, our understanding of the functional role of MPC1 is currently confined to *in vitro* studies and has yet to be further explored *in vivo* to ascertain its impact on the development of PH. Moreover, the use of α-SMA as a marker for PASMCs, while widely recognized and accepted, introduces a degree of nonspecificity due to its potential expression in other cell types, such as fibroblasts. This may adversely affect the accurate identification and analysis of PASMCs. Furthermore, since specific inhibitors of LONP1 have not yet been developed, the use of BTZ in this study may have introduced nonspecific effects, potentially compromising the accuracy of the results. Lastly, the inherent difficulty in obtaining lung tissue specimens from PH patients has resulted in a lack of data support from human PH samples in this study.

## Conclusion

**Figure 9:**
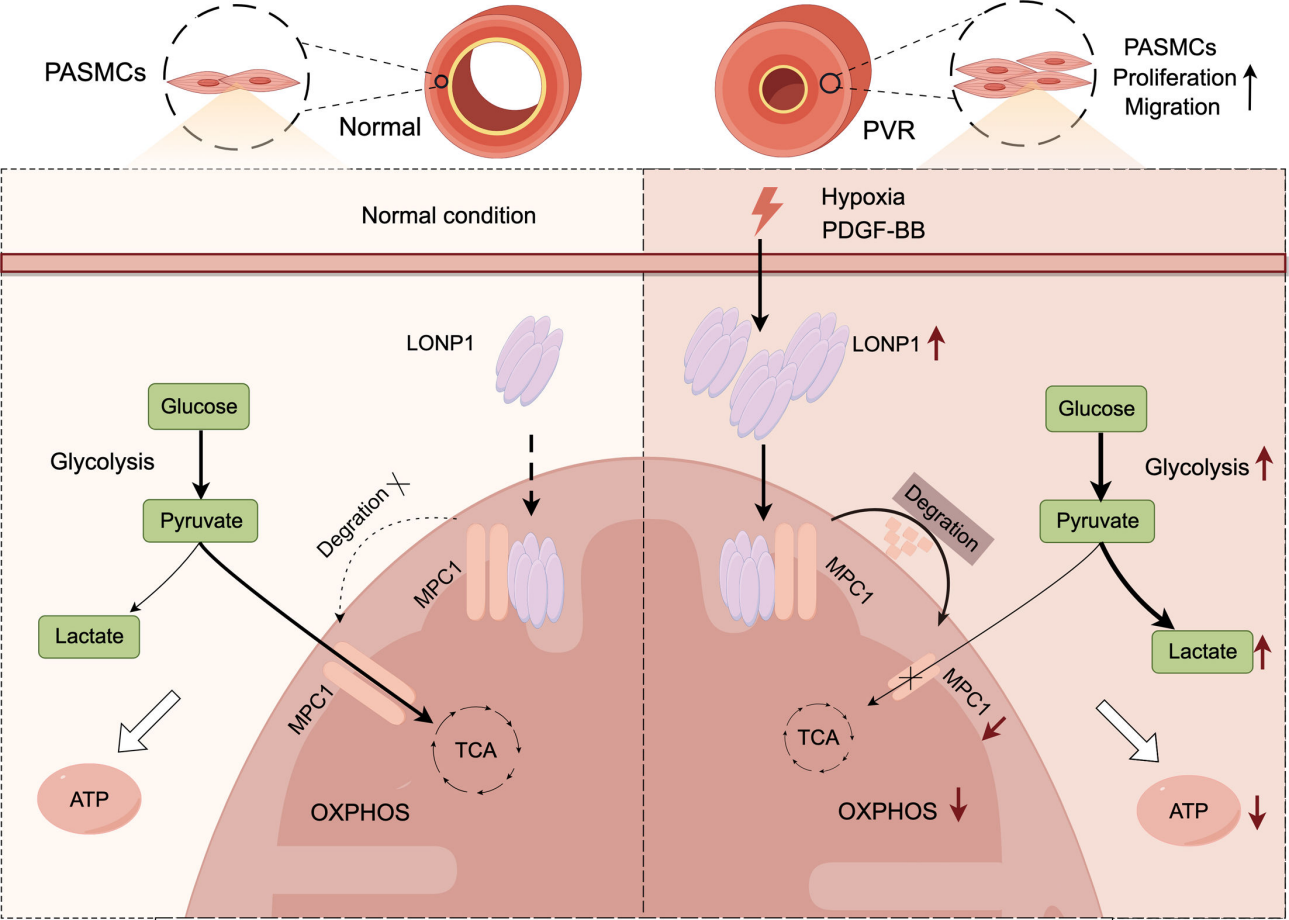
The schematic diagram illustrates the regulatory mechanism of LONP1 in PH. Pathogenic factors promote the up-regulation of LONP1 expression, causing LONP1 to directly interact with and degrade MPC1. This leads to the inability of pyruvate in the cytoplasm to enter mitochondria for subsequent OXPHOS, resulting in more pyruvate being directed toward the glycolysis pathway to produce lactate. This glycolytic reprogramming promotes the proliferation and migration of PASMCs, ultimately contributing to PVR. The image was created by Figdraw.com. LONP1, Lon protease 1; MPC1, mitochondrial pyruvate carrier 1; OXPHOS, oxidative phosphorylation; PASMCs, pulmonary artery smooth muscle cells; PH, pulmonary hypertension; PVR, pulmonary vascular remodeling.

Our study indicates that up-regulated LONP1 mediates PVR in PH, and inhibiting LONP1 is sufficient to effectively protect rats from PH. Mechanistically, LONP1 promotes glycolytic reprogramming, proliferation, and migration of PASMCs by degrading MPC1, ultimately leading to PVR (Figure 9). Therefore, targeting LONP1 holds promise as a potential therapeutic strategy for PH.

**Clinical Perspectives**

- Glycolytic reprogramming plays a pivotal role in the pathogenesis of pulmonary hypertension (PH); however, the underlying mechanisms triggering glycolytic reprogramming in pulmonary artery smooth muscle cells (PASMCs) require further elucidation.
- Lon protease 1 (LONP1) promoted glycolytic reprogramming, proliferation, and migration of PASMCs by degrading mitochondrial pyruvate carrier 1 (MPC1). LONP1 knockdown prevented monocrotaline (MCT)-induced PH in rats. Therapeutically, knockdown or pharmacological inhibition of LONP1 significantly reversed MCT-induced PH in rats.
- Targeting LONP1 may represent a promising therapeutic strategy for PH.

## Supplementary material

Online supplementary figure S1-S12

## Data Availability

The data that support the findings of the present study are available from the corresponding author on reasonable request.

## Abbreviations

AAV: adeno-associated virus
DAPI: 4’,6-diamidino-2-phenylindole
GLUT1: glucose transporter-1
LDHA: lactate dehydrogenase A
LONP1: Lon protease 1
MCT: monocrotaline
MPC1: mitochondrial pyruvate carrier 1
PASMCs: pulmonary artery smooth muscle cells
PAT: pulmonary artery acceleration time
PCNA: proliferating cell nuclear antigen
PDGF-BB: platelet-derived growth factor BB
PET: pulmonary artery ejection time
PH: pulmonary hypertension
PVR: pulmonary vascular remodeling
TAPSE: tricuspid annular plane systolic excursion
qPCR: quantitative real-time polymerase chain reaction
