# Supplementary material for: LONP1 facilitates pulmonary artery smooth muscle cell glycolytic reprogramming by degrading MPC1 in pulmonary hypertension
Source: Clin Sci (Lond). 2025 May 20;139(10):479–501. doi: 10.1042/CS20255922 (PMC12203999; doi:10.1042/CS20255922)
Supplement: Online supplementary figure S1-S12 [file CS-139-10-CS20255922-s001.docx]

**Supplementary material**


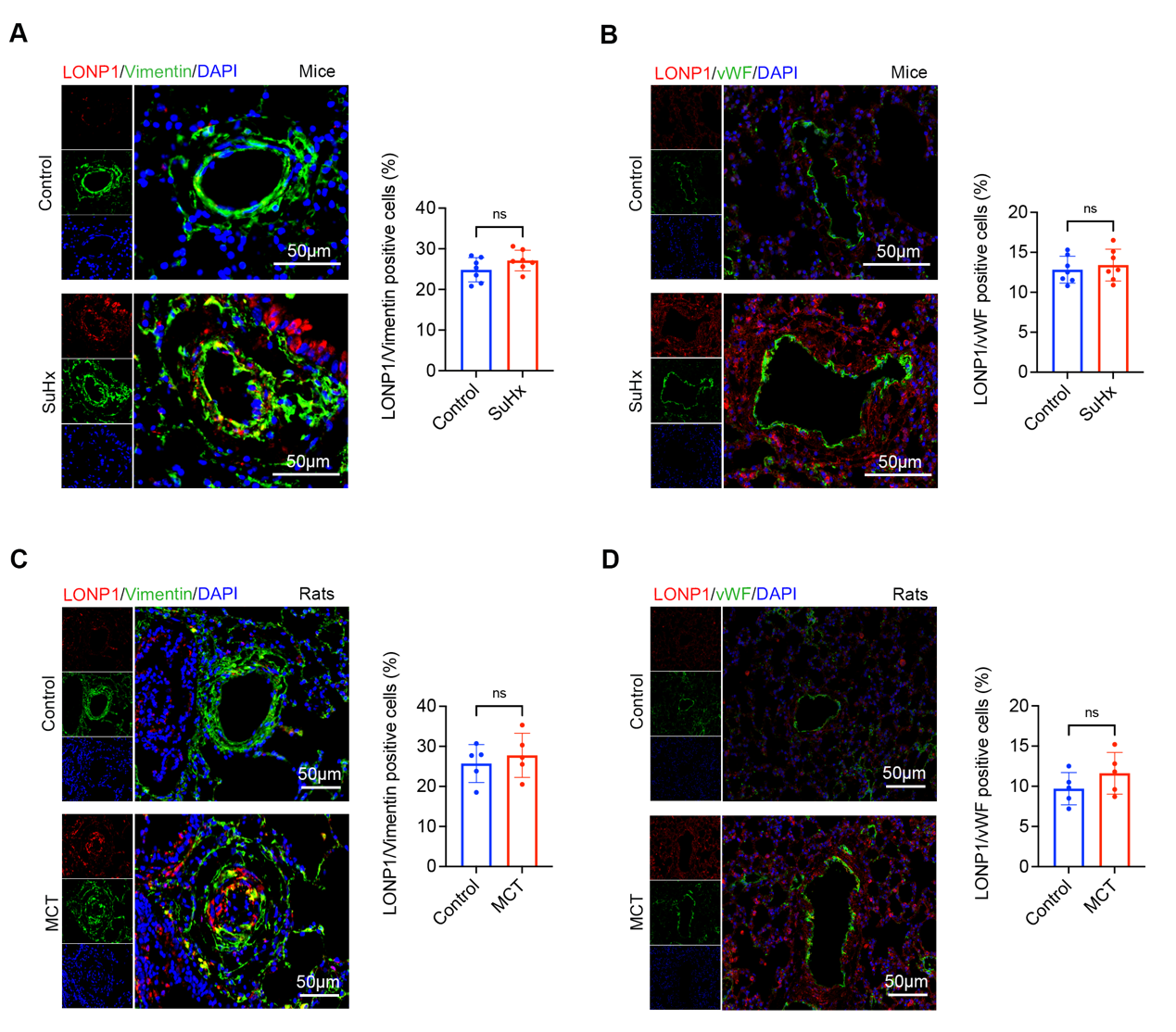


Supplementary Figure 1. ﻿Expression of LONP1 in fibroblasts and endothelial cells of PH mice and rats. A. Representative IF staining of LONP1 (red) and vimentin (green) and quantitative analysis in lung sections from control and SuHx-induced mice (n=7). B. Representative IF staining of LONP1 (red) and vWF (green) and quantitative analysis in lung sections from control and SuHx-induced mice (n=7). C. Representative IF staining of LONP1 (red) and vimentin (green) and quantitative analysis in lung sections from control and MCT-induced rats (n=5). D. Representative IF staining of LONP1 (red) and vWF (green) and quantitative analysis in lung sections from control and MCT-induced rats (n=5). Nuclei were stained with DAPI (blue). Scale bar = 50 µm. Data are presented as mean ± SD. Student’s t-test was used for comparisons between two groups. **P* < 0.05, ***P* < 0.01, ****P* < 0.001.


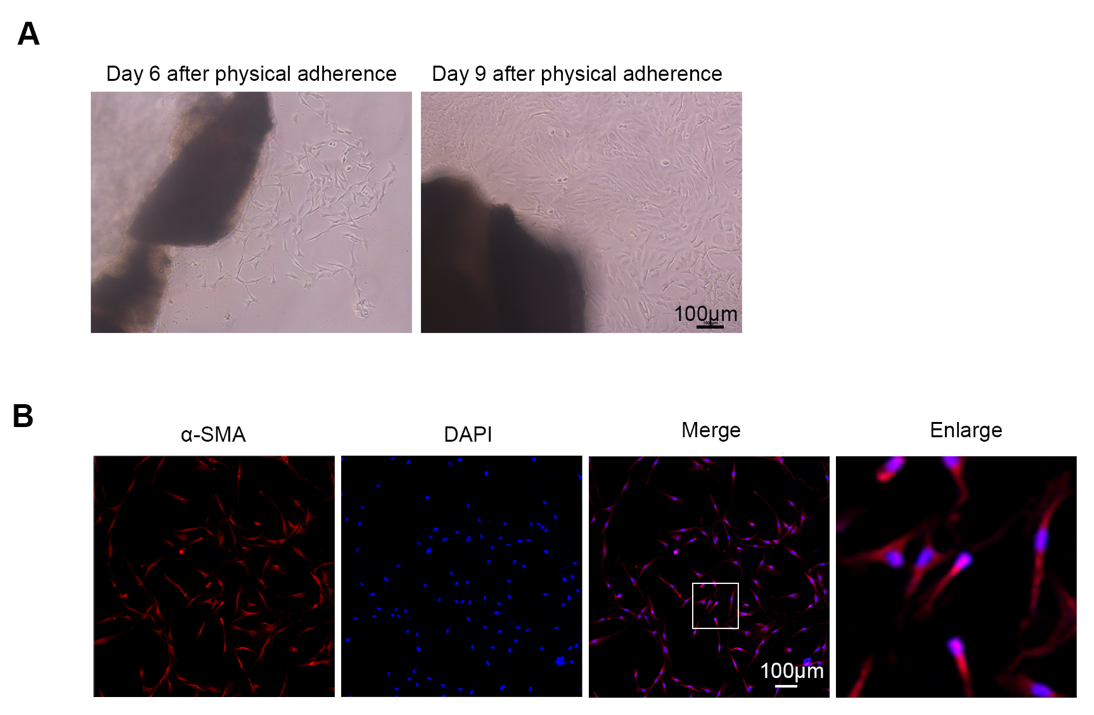


Supplementary Figure 2. Culture and identification of primary PASMCs from rats. A. PASMCs migrated out from the adherent pulmonary artery fragments. Scale bar = 100 µm. B. Representative IF staining of α-SMA (red) in the second passage cells. Nuclei were stained with DAPI (blue). Scale bar = 100 µm.


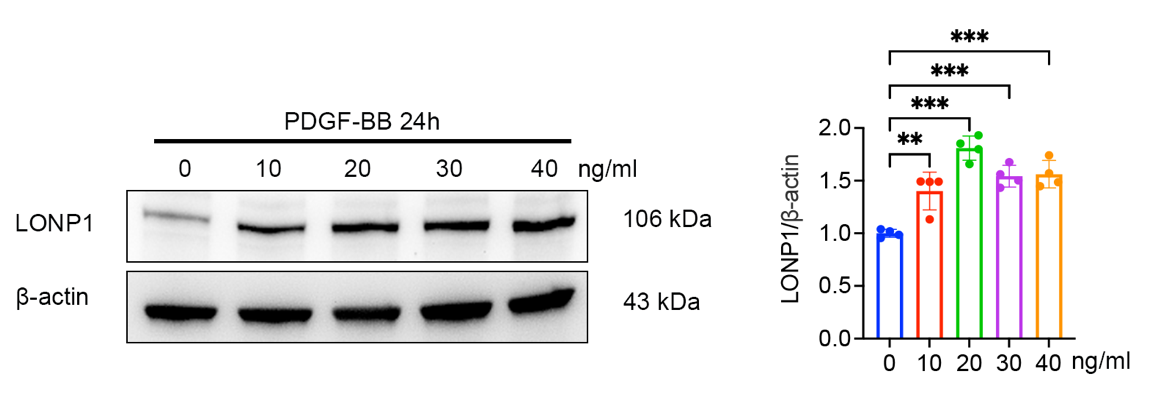


Supplementary Figure 3. PDGF-BB stimulated a concentration-dependent increase in LONP1 expression. Representative Western blot and quantitative analysis of LONP1 protein expression in PASMCs stimulated with various concentrations of PDGF-BB for 24 hours (n=4). Data are presented as mean ± SD. One-way ANOVA ﻿with Bonferroni multiple comparisons test was used for comparisons among multiple groups. **P* < 0.05, ***P* < 0.01, ****P* < 0.001.


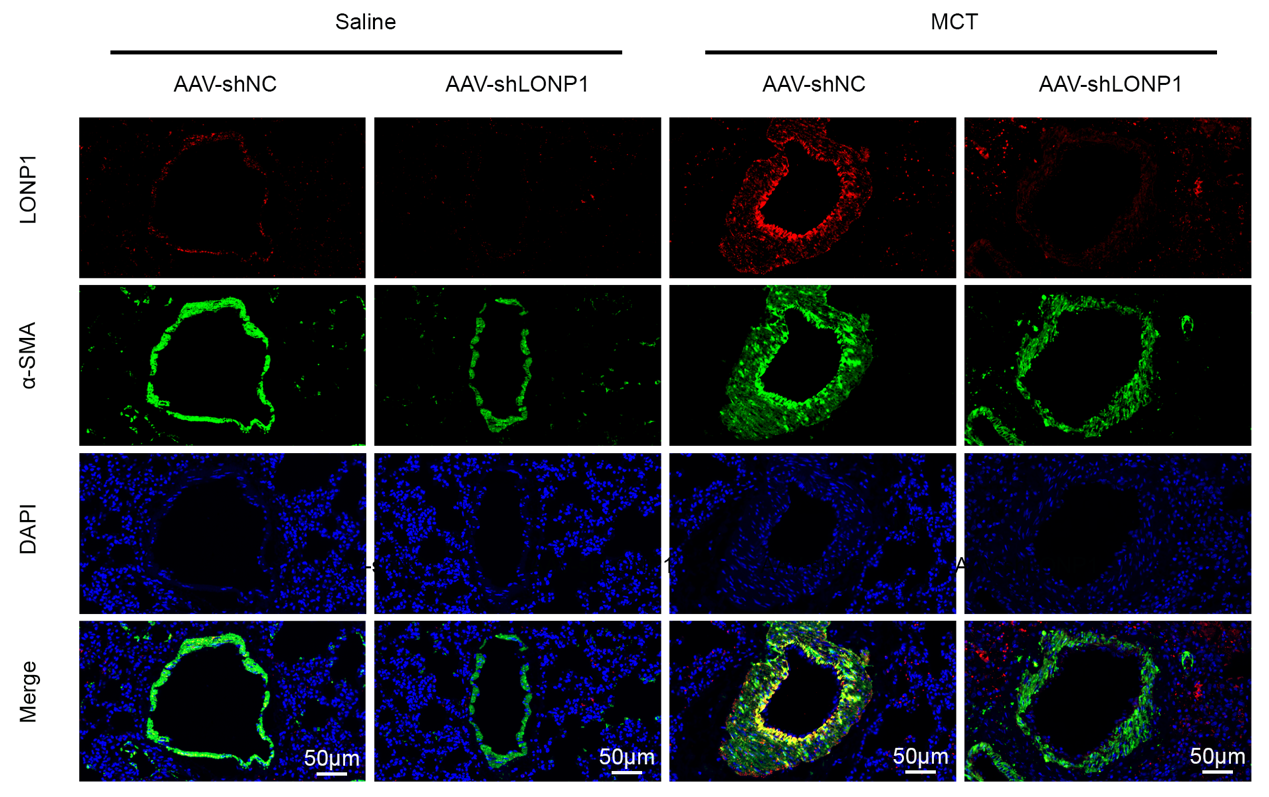


Supplementary Figure 4. IF staining revealed a significant reduction in LONP1 protein expression in pulmonary arteries following AAV-shLONP1 delivery, confirming the knockdown efficiency of AAV-shLONP1. Representative IF staining of LONP1 (red) and α-SMA (green) in rat lung sections. Nuclei were stained with DAPI (blue). Scale bar = 50 µm.


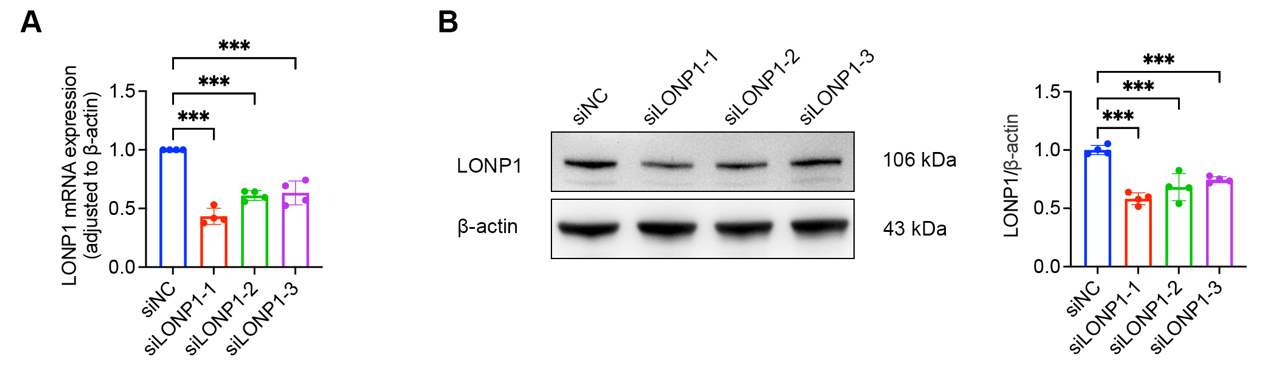


Supplementary Figure 5. Efficiency confirmation of siRNA-mediated knockdown of LONP1 in PASMCs. Based on the knockdown efficiency, siLONP1-1 was selected for the subsequent experiments. A. qPCR analysis of LONP1 mRNA expression in PASMCs (n=4). B. Representative Western blot and quantitative analysis of LONP1 protein expression in PASMCs (n=4). Data are presented as mean ± SD. One-way ANOVA ﻿with Bonferroni multiple comparisons test was used for comparisons among multiple groups. **P* < 0.05, ***P* < 0.01, ****P* < 0.001.


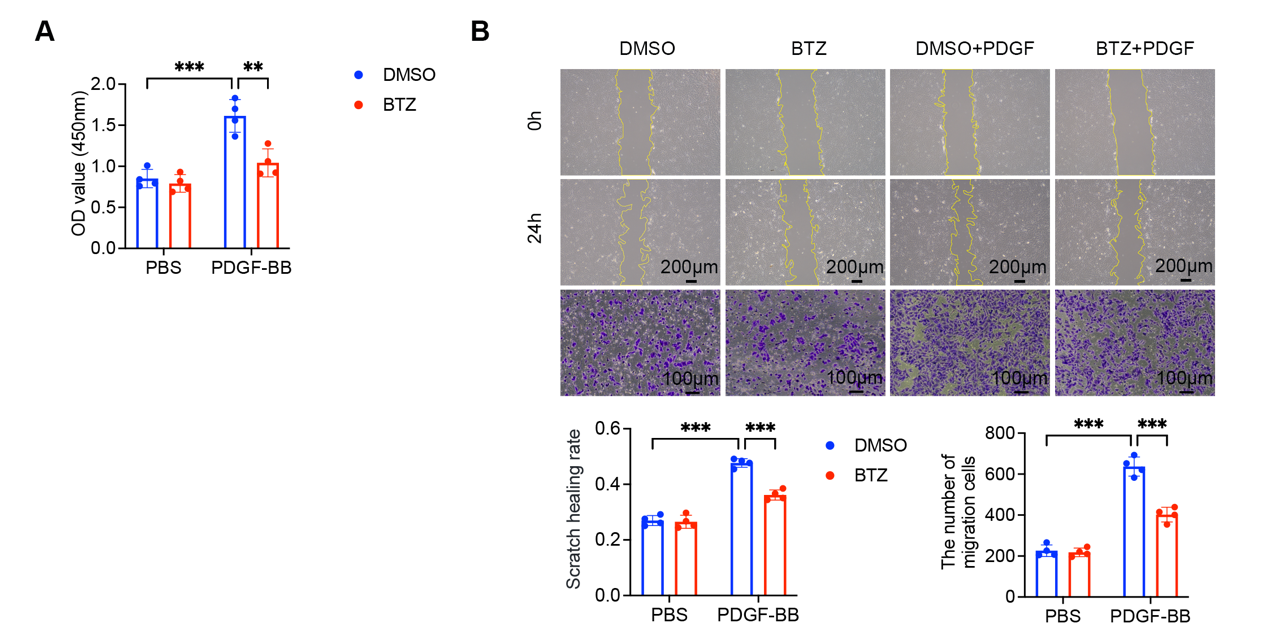


Supplementary Figure 6. BTZ significantly inhibited the increased cell viability and migration capacity of PASMCs under PDGF-BB treatment. A. Cell viability was assessed by CCK-8 assay at 450 nm absorbance (n=4). B. Representative images and quantitative analysis of wound healing and Transwell assays (n=4). Scale bar = 200 µm for wound healing assay. Scale bar = 100 µm for Transwell assay. Data are presented as mean ± SD. Two-way ANOVA ﻿with Bonferroni multiple comparisons test was used for comparisons among multiple groups. **P* < 0.05, ***P* < 0.01, ****P* < 0.001.


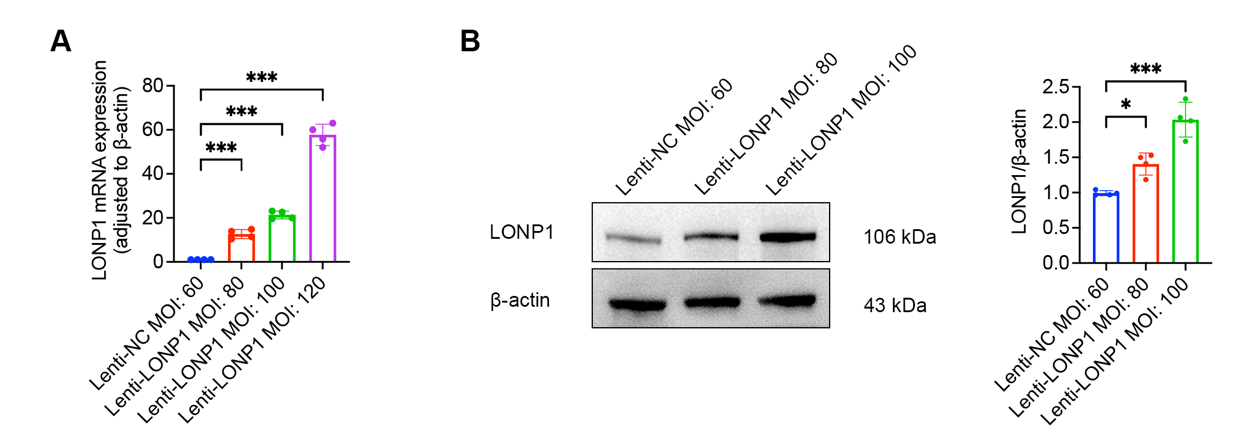


Supplementary Figure 7. Efficiency confirmation of lentivirus-mediated overexpression of LONP1 in PASMCs. For the subsequent experiments, a multiplicity of infection (MOI) of 60 was selected for Lenti-NC, and an MOI of 100 was selected for Lenti-LONP1. A. qPCR analysis of LONP1 mRNA expression in PASMCs (n=4). B. Representative Western blot and quantitative analysis of LONP1 protein expression in PASMCs (n=4). Data are presented as mean ± SD. One-way ANOVA ﻿with Bonferroni multiple comparisons test was used for comparisons among multiple groups. **P* < 0.05, ***P* < 0.01, ****P* < 0.001.


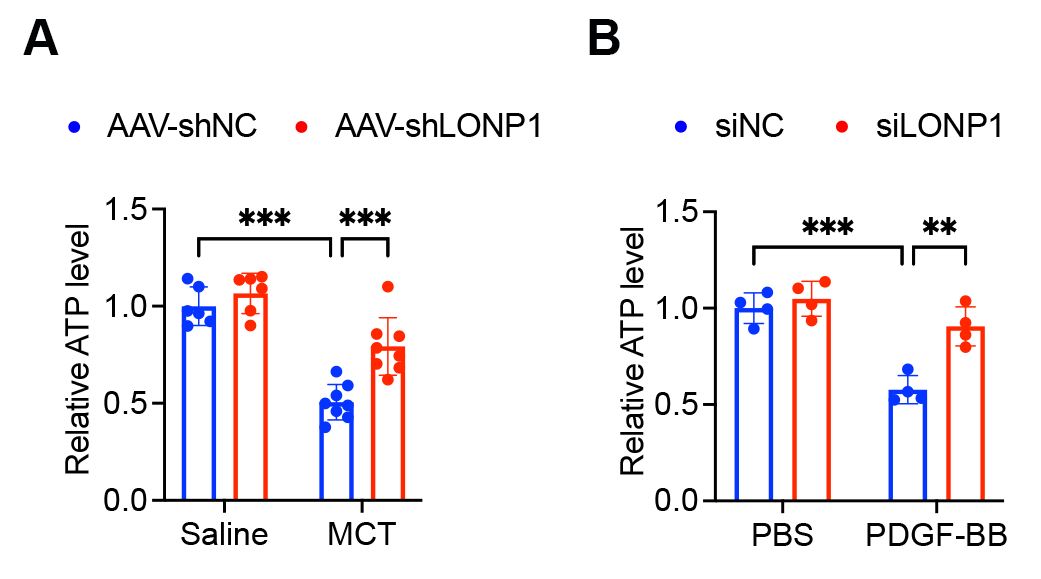


Supplementary Figure 8. The ATP levels were detected by using a commercial kit. A. Relative ATP levels in pulmonary artery homogenates from the AAV-shNC, AAV-shLONP1, AAV-shNC+MCT, and AAV-shLONP1+MCT groups (n=6-8). B. Relative ATP levels in PASMCs transfected with siNC or siLONP1, with or without 20 ng/ml PDGF-BB stimulation for 24 hours (n=4). Data are presented as mean ± SD. Two-way ANOVA ﻿with Bonferroni multiple comparisons test was used for comparisons among multiple groups. **P* < 0.05, ***P* < 0.01, ****P* < 0.001.


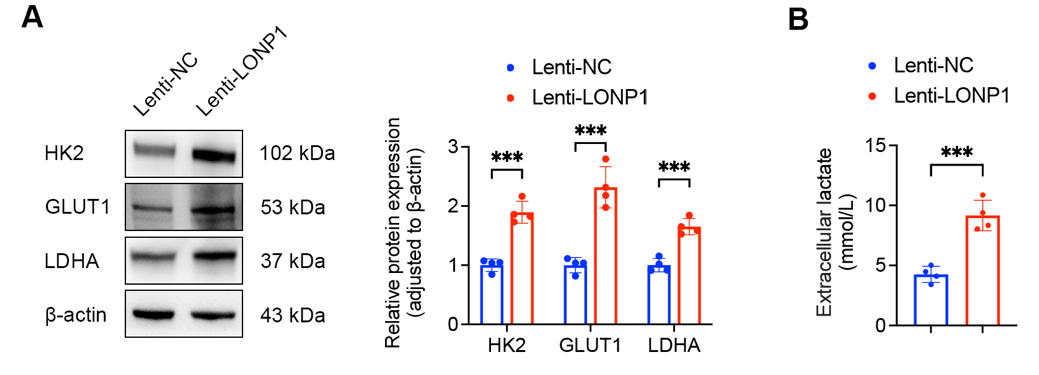


Supplementary Figure 9. Overexpression of LONP1 enhances glycolysis in PASMCs. A. Representative Western blot and quantitative analysis of HK2, GLUT1, and LDHA protein expression in PASMCs transfected with Lenti-NC and Lenti-LONP1 (n=4). B. Lactate levels in the supernatants of PASMC cultures from each group (n=4). Data are presented as mean ± SD. Student’s t-test was used for comparisons between two groups. **P* < 0.05, ***P* < 0.01, ****P* < 0.001.


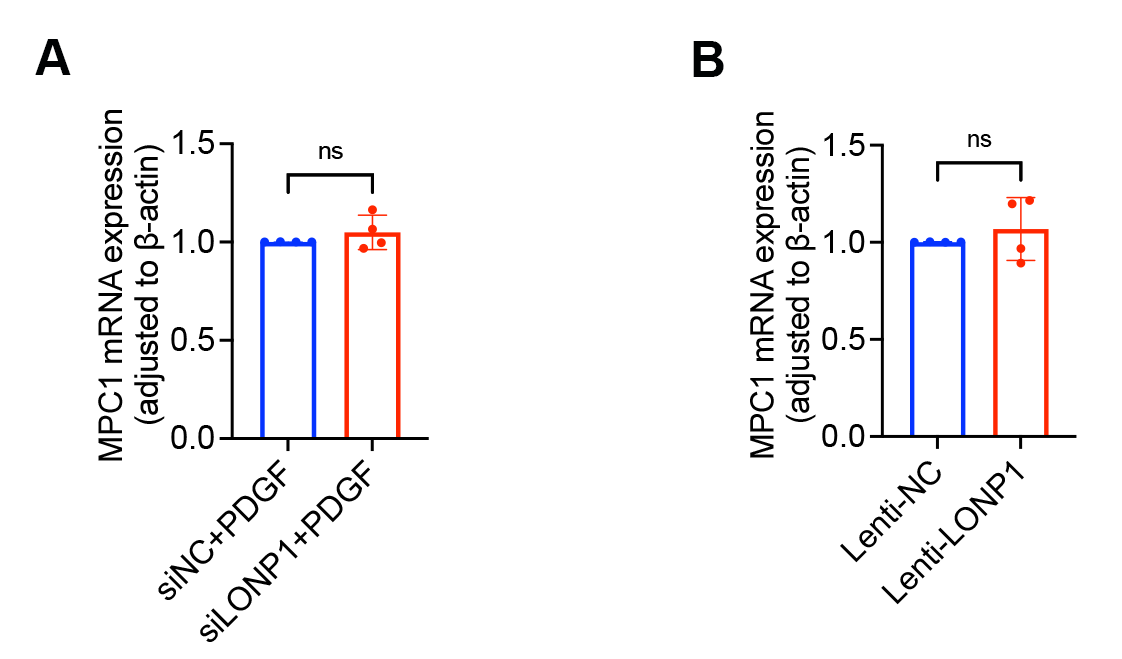


Supplementary Figure 10. MPC1 mRNA expression remained consistent regardless of LONP1 knockdown or overexpression. qPCR analysis of MPC1 mRNA expression in PASMCs with LONP1 knockdown (A) or overexpression (B) (n=4). Data are presented as mean ± SD. Student’s t-test was used for comparisons between two groups.


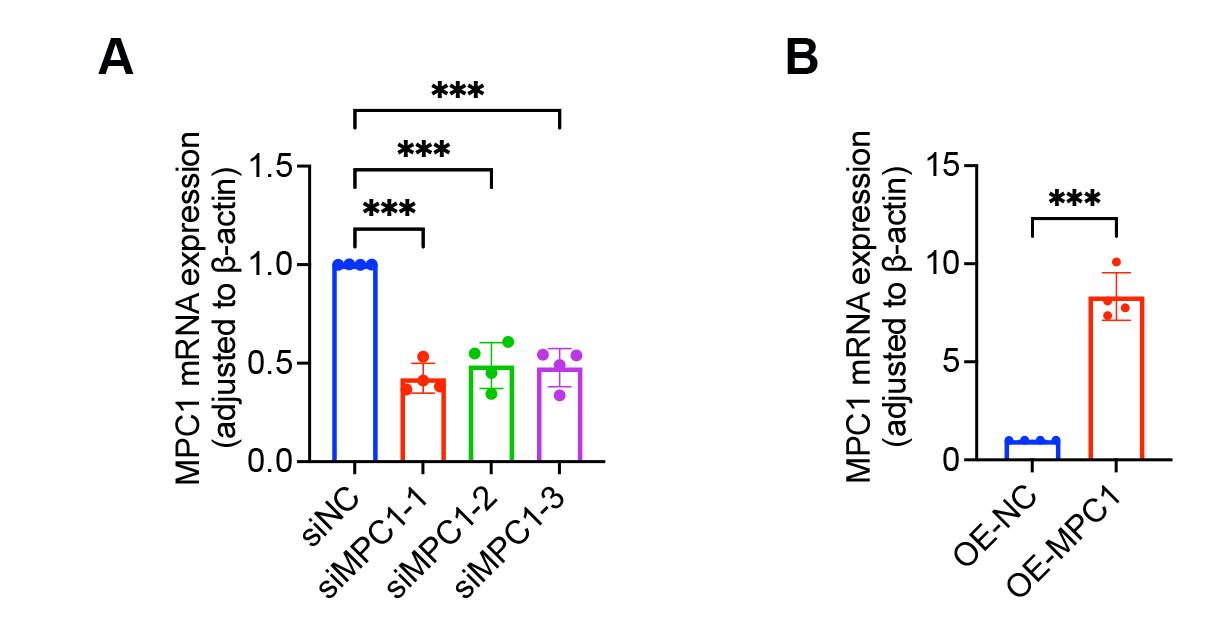


Supplementary Figure 11. Efficiency confirmation of siRNA-mediated knockdown of MPC1 and plasmid-mediated overexpression of MPC1 in PASMCs. Based on the knockdown efficiency, siMPC1-1 was selected for the subsequent experiments. A. qPCR analysis of MPC1 mRNA expression in PASMCs transfected with either siNC or siMPC1 (n=4). B. qPCR analysis of MPC1 mRNA expression in PASMCs transfected with either OE-NC or OE-MPC1 plasmids (n=4). Data are presented as mean ± SD. Student’s t-test was used for comparisons between two groups, and one-way ANOVA with Bonferroni multiple comparisons test was used for comparisons among multiple groups. **P* < 0.05, ***P* < 0.01, ****P* < 0.001.


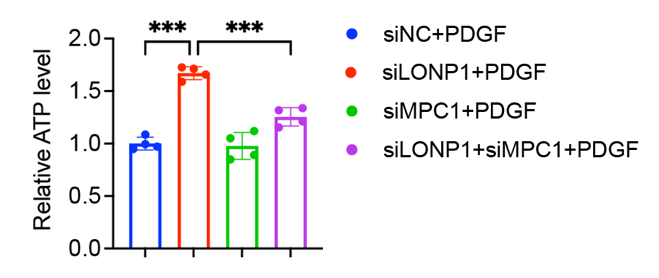


Supplementary Figure 12. Relative ATP levels in PASMCs transfected with siNC, siLONP1 or (and) siMPC1, with 20 ng/ml PDGF-BB stimulation for 24 hours (n=4). Data are presented as mean ± SD. One-way ANOVA with Bonferroni multiple comparisons test was used for comparisons among multiple groups. **P* < 0.05, ***P* < 0.01, ****P* < 0.001.
